# Meter‐Scale Ultra‐Large‐Area Flexible Electroluminescent Devices Enabled by Aerosol Spraying

**DOI:** 10.1002/advs.202523831

**Published:** 2026-01-26

**Authors:** Hao Song, Liang Yi, Lixiang Huang, Wajid Ali, Linhan Cai, Chao Wang, Jianhua Huang, Ziwei Li, Anlian Pan

**Affiliations:** ^1^ Hunan Institute of Optoelectronic Integration College of Materials Science and Engineering Hunan University Changsha China; ^2^ Changsha Blue Ink Technology Co., Ltd Changsha China; ^3^ School of Physics and Electronics Hunan Normal University Changsha China

**Keywords:** electroluminescence, fluorescence material, large‐scale display, light–emitting device, ZnS:Cu

## Abstract

Flexible alternating current electroluminescent (ACEL) devices have garnered significant research interest due to their potential applications in illumination and display. However, challenges remain in fabricating ACEL devices with high brightness and excellent working stability over a large luminescent area. In this study, we demonstrate a simple aerosol spraying method with modified luminescent ink to fabricate meter‐scale flexible ACEL devices. In the fabrication process, the particle dispersibility of luminescent ink and the uniformity of sprayed film have been significantly improved. This improvement is primarily attributed to the introduction of long‐chain molecular groups of the surfactant, which can bind to the surface of ZnS:Cu luminescent particles at one end, while the other end remains suspended in the oily solvent, providing substantial steric hindrance. The fabricated planar thin‐film ACEL devices demonstrated excellent substrate compatibility and flexibility, with exceptional operational stability in high‐temperature, humid, and water‐rich environments. Remarkably, we achieved a maximum brightness of nearly 500 cd·m^−2^ in small‐sized devices, while a brightness of 303.3 cd m^−2^ from an ultra‐large device of 1.2 m × 1.2 m. These devices have been further explored as navigation signs for autonomous takeoff and landing of industry drones.

## Graphical abstract

This work presents a simple aerosol spraying method using modified luminescent ink to fabricate meter‐scale flexible alternating current electroluminescent (ACEL) devices. Enhanced particle dispersibility and film uniformity yield impressive brightness up to 500 cd m^−2^, positioning these devices as effective luminous navigation signs for industrial drones.

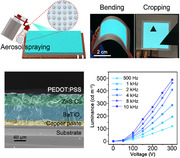

## 1 Introduction

Visual displays, as a core component, have become the primary medium for information dissemination. The growing demand for these displays has significantly accelerated the development of electronic display technologies, from traditional liquid crystal displays (LCDs) and light–emitting diodes (LEDs), into thin‐film light–emitting devices, involving organic light–emitting diodes (OLEDs), quantum dot light–emitting diodes (QLEDs), and alternating current electroluminescent (ACEL) [1, 2, 3]. Traditional non‐self‐emissive LCDs, known for their high stability, resolution, and cost‐effectiveness, continue to dominate modern display technology [4, 5]. However, their device structure, which includes LED backlight sources, liquid crystal layers, and color filters, results in bulky volumes and rigid substrates, significantly restricting their adoption in flexible display applications [2, 6]. Besides, self‐emissive displays, such as OLED, QLED, and micro‐LED, have made significant progress, offering remarkable advantages in broader color gamut and enhanced flexibility [7, 8]. Despite this, these technologies still face challenges, including the inherent instability of luminescent materials, limited light emission brightness, and the need for precise manufacturing conditions [9, 10].

In contrast, ACEL devices, with their simple manufacturing process, excellent mechanical flexibility, and long lifespan, have emerged as strong contenders for flexible displays [3, 11]. The large‐area and uniform fabrication of ACEL devices is essential for their practical application in solid‐state lighting and flexible displays [12, 13]. Traditional spin‐coating methods rely on centrifugal force to spread the liquid from the center of the substrate to its edges [14]. However, as the area increases, the centrifugal force during rotation leads to uneven solute distribution, limiting the size of the resulting light–emitting devices to only a few square millimeters to square centimeters [15, 16]. Methods such as doctor‐blade coating or screen printing are hindered by the narrow depth of the coating device (typically within a few micrometers), which causes surface height variations in flexible substrates, leading to scratches or significant thickness fluctuations in the luminescent film [13, 17]. Inkjet printing, a non‐contact liquid film‐forming technology, faces challenges due to the complex fluid and evaporation dynamics of the ink, often causing the coffee‐ring effect during droplet drying, which complicates large‐area uniform preparation of luminescent film [18, 19].

The common challenge across the above existing techniques in achieving large‐area uniform pattern preparation is the uniform dispersion of functional particles in the ink, and the uniform diffusion of droplets during the film‐forming process [20, 21]. In comparison, aerosol spraying, as a solution atomization deposition technique, atomizes the ink into fine mist‐like droplets under pressure within the spray can, which can adhere to various substrates [22, 23]. The aerosolized functional inks are beneficial for large‐area uniform film preparation and offer advantages such as low material waste, functional ink compatibility, and roll‐to‐roll technology compatibility [15, 24]. It is expected to resolve the issues in spin‐coating, doctor‐blading, and other processes. Despite the inherent advantages of aerosol spraying, the development of highly dispersed functional ink materials remains a significant challenge in this field. Additionally, large‐area ACEL devices with the meter‐scale size have been rarely reported.

Herein, a simple fully‐sprayed method using modified luminescent ink is proposed to realize the uniform fabrication of ultra‐large‐area ACEL devices. The uniformity of the luminescent particles in the ink and the luminescent film after spraying was significantly improved, by introducing surface chemical modification of ZnS:Cu luminescent particles with the functional surfactant of sodium fatty alcohol polyoxyethylene ether sulfate (AES). The hydrophilic hydroxyl groups on the surface of ZnS:Cu particles interact with the sulfate groups of AES molecules, forming hydrophobic and oleophilic long‐chains allowing for better dispersion in oily solvents. This effectively inhibits particle agglomeration by increasing the spatial steric hindrance of particles in the ink. Our aerosol‐sprayed planar thin‐film ACEL devices exhibit an ultrahigh luminous brightness up to 500 cd m^−2^, along with excellent substrate compatibility and extreme environmental stability. For the first time, we report achieving an ultra‐large‐area ACEL device of 1.2 m × 1.2 m with a remarkable brightness of 303.3 cd m^−2^. Simulations highlight the crucial role of highly dispersed luminescent particles in achieving high‐brightness devices. This ultra‐large‐area ACEL device has been developed as a night‐landing navigation platform for industrial drone delivery applications.

## 2 Results and Discussion

**FIGURE 1:**
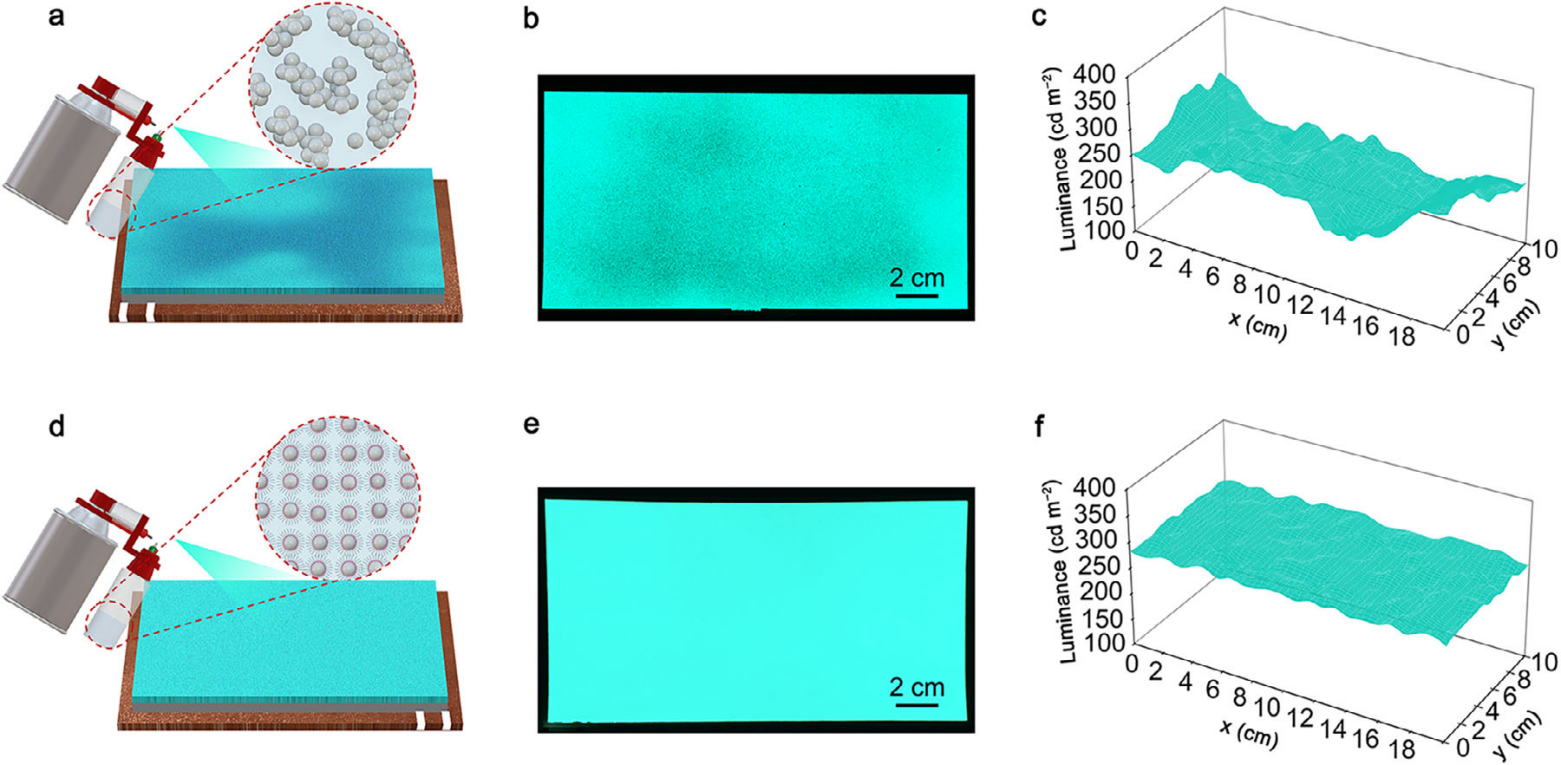
Advantages of aerosol spraying modified luminescent ink for producing large‐area ACEL devices. (a) Schematics of spraying the original ink to make ACEL device. (b) Photograph of the as‐prepared device with original ink, exhibiting uneven distribution of luminous intensity. (c) Luminance mapping of the ACEL device. (d) Schematics of spraying modified luminescent ink to make ACEL device. (e) Photograph of the as‐prepared device with modified luminescent ink, demonstrating uniform distribution of luminous intensity. (f) Luminance mapping of the as‐prepared ACEL device using modified luminescent ink. The luminescent area of devices is 10 × 20 cm^2^, and the AC voltage is set at 300 V with a frequency of 2 kHz.

Figure 1a,b present schematics of the aerosol spraying process used to fabricate large‐area ACEL devices with both original and modified ZnS:Cu luminescent inks, respectively. From the inset images, it is evident that the original luminescent particles are randomly aggregated into large clusters within the ink, whereas this aggregation is completely mitigated in the modified luminescent ink, which exhibits a uniform dispersion. Figure 1b shows a photograph of the working ACEL device fabricated using original inks, which exhibits inhomogeneous luminance distribution due to the uncontrolled aggregation of luminescent particles. The mapping image indicates significant fluctuations in luminance, with maximum and minimum values of 280 and 175 cd m^−2^, respectively (Figure 1c). Here, the AC voltage of devices was set at 300 V with a frequency of 2 kHz. In contrast, Figure 1e clearly shows that homogeneous luminance can be achieved using the modified luminescent ink. Benefitting from the excellent dispersibility of luminescent particles in the improved ink, the ACEL device demonstrates uniform electroluminescent brightness within a luminescent area of 10 × 20 cm^2^, while the corresponding luminance mapping exhibits only slight fluctuations in the range of 270–280 cd m^−2^ (Figure 1f).

**FIGURE 2:**
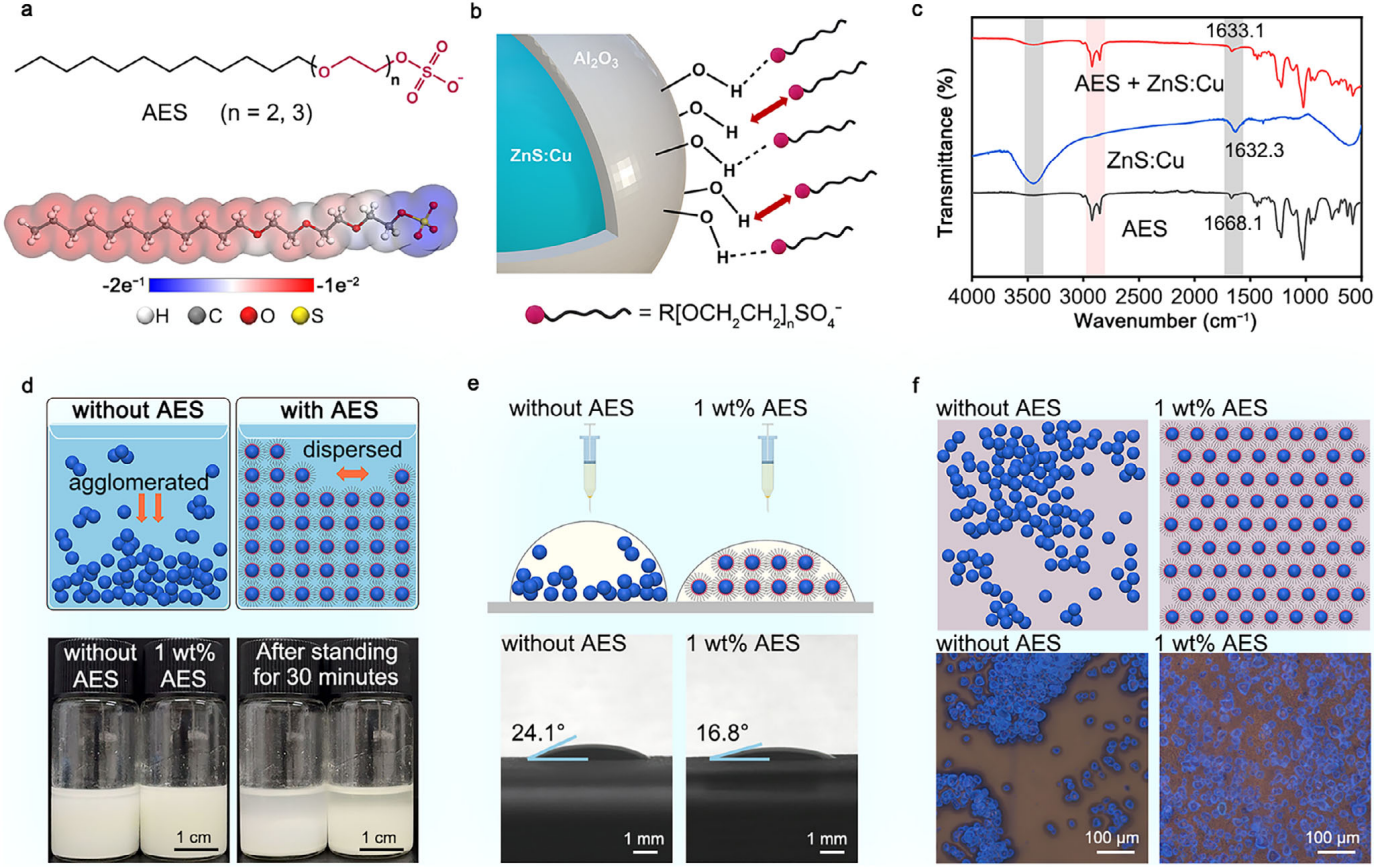
Chemical structure and characterization of modified luminescent inks. (a) Molecule structure and ESP map of AES molecules. (b) Schematics of a ZnS:Cu luminescent particle bonded with AES surface ligands. (c) FTIR spectra of AES, untreated ZnS:Cu particles, and AES modified ZnS:Cu particles. (d) Top: Schematics of particle dispersity in untreated and modified inks. Bottom: Photos of untreated ink (without AES) and modified ink (with 1 wt.% AES) before and after standing for 30 min. (e) Top: Schematics of particle distribution in droplets of untreated and modified inks. Bottom: Comparison of contact angle test. (f) Top: Schematics of particle distributions sprayed on the substrate. Bottom: The comparison of corresponding fluorescence microscopy images.

To achieve the fabrication of large‐scale ACEL device, the main challenge lies in optimizing the dispersion of the fluorescent particles in the ink, which further affects the spraying uniformity of the light–emitting layer. The detailed synthesis of original and modified luminescent inks of ZnS:Cu particles can be found in Methods. Figure 2a illustrates the chemical structure of AES and its electrostatic potential (ESP) map. The molecule consists of a long‐chain alkyl hydrophobic backbone, polyethylene glycol ether segments (*n* = 2–3), and terminal sulfate anion hydrophilic groups (−SO_4_^−^). The charge distribution diagram clearly shows that the chlorine and sulfur atoms at the end of the molecular chain exhibit strong electronegativity.

Considering that the surface of ZnS:Cu fluorescent particles in inks is coated with a thin passivation shell of aluminum oxide (AlO_x_) containing abundant short chain of −OH groups (Methods; Section S1), which exhibit strong polarity and hydrophilicity, creating a significant surface energy disparity when immersed in the oily ink [25]. This causes the particles tend to agglomerate to minimize the overall surface energy, in accordance with the thermodynamic principles. Furthermore, the network of strong hydrogen bonds formed between the surface hydroxyl groups provides an additional driving force that promotes further agglomeration of the particles in the luminescent ink. Such agglomeration may significantly compromise the coating uniformity of the luminescent layer after spraying.

Figure 2b illustrates the schematics of preventing particle agglomeration in the ink through surface modification. Upon introducing AES into the initial ZnS:Cu fluorescent ink at an appropriate concentration and under controlled processing conditions, the terminal −SO_4_^−^ anionic group, exhibiting strong electronegativity, interacts with the hydroxyl groups on the particle surface. This interaction leads to the formation of stable chemical bonds, anchoring long‐chains of AES onto the particle surface and creating a dense steric hindrance layer that ensures the uniform dispersion of luminescent particles in oil solvent. As a result, when adjacent particles approach each other, the overlapping and entanglement of long chains effectively prevent particle agglomeration.

Figure 2c displays and compares the Fourier‐transform infrared (FTIR) spectra of AES molecules, untreated ZnS:Cu powders, and modified ZnS:Cu powders. AES molecules exhibit several distinctive peaks around 2900 cm^−1^ [26]. The untreated ZnS:Cu powders show characteristic stretching vibrations at 3452.4 cm^−1^ and bending vibrations at 1632.2 cm^−1^, originating from hydroxyl groups on the AlO_x_ surface [27]. However, in the modified ZnS:Cu particles, the characteristic stretching and bending vibration peaks of ZnS:Cu are significantly weakened and shifted. Additionally, a few characteristic peaks around 2900 cm^−1^, consistent with the AES molecule appears in the modified ZnS:Cu. These experimental results directly confirm that new chemical bonding have occurred on the particle surface, and the AES molecules have affected the molecular vibrational characteristics of the ZnS:Cu particles.

To achieve optimal particle dispersion in the ink during subsequent spraying processes, the effect of AES molecular concentration on the luminescent film uniformity has been systematically investigated and characterized. Our experiments reveal that the ink viscosity significantly decreases with increasing concentration of AES molecules (Section S2). However, excessive addition of AES can cause a decline in PL lifetime and PLQY of ink (Section S3). This suggests that an appropriate concentration can balance good rheological properties and luminescent performance. Based on the comprehensive consideration of luminescent property liquid and flow rate, the optimal AES concentration in our experiments was determined to be 1 wt.%.

Figure 2d presents schematics of particle dispersion in untreated ink and 1 wt.%‐AES‐modified ink. The bottom photographs display the experimental results of the dissolution and sedimentation states of two luminescent inks, both immediately after synthesis and after 30 min of standing. The untreated ink with uncontrollable aggregation of fluorescent particles, shows a noticeable sedimentation after 30 min of standing. In contrast, the modified ink demonstrates improved particle dissolution with reduced sedimentation after 30 min. Notably, the turbidity of the modified solution increases slightly, indicating enhanced solubility of the luminescent particles. Moreover, it is also essential to consider the wettability of the ink on the substrate layers and the coffee ring effect associated with the solvent evaporation process. Figure 2e compares the wettability of spray droplets from untreated and AES‐modified inks on the dielectric layer. Contact‐angle experiments revealed that the untreated ink droplets and the modified ink droplets exhibited contact angles of 24.1° and 16.8°, respectively. The lower contact angle of the AES‐modified ink suggests enhanced wettability, which facilitates the formation of a more uniform film after spreading. Figure 2f presents fluorescence microscope images of luminescent ink sprayed onto a substrate using these two inks. After drying, the AES‐modified ink showed no significant aggregation, resulting in a more uniform dispersion within a thin‐film.

**FIGURE 3:**
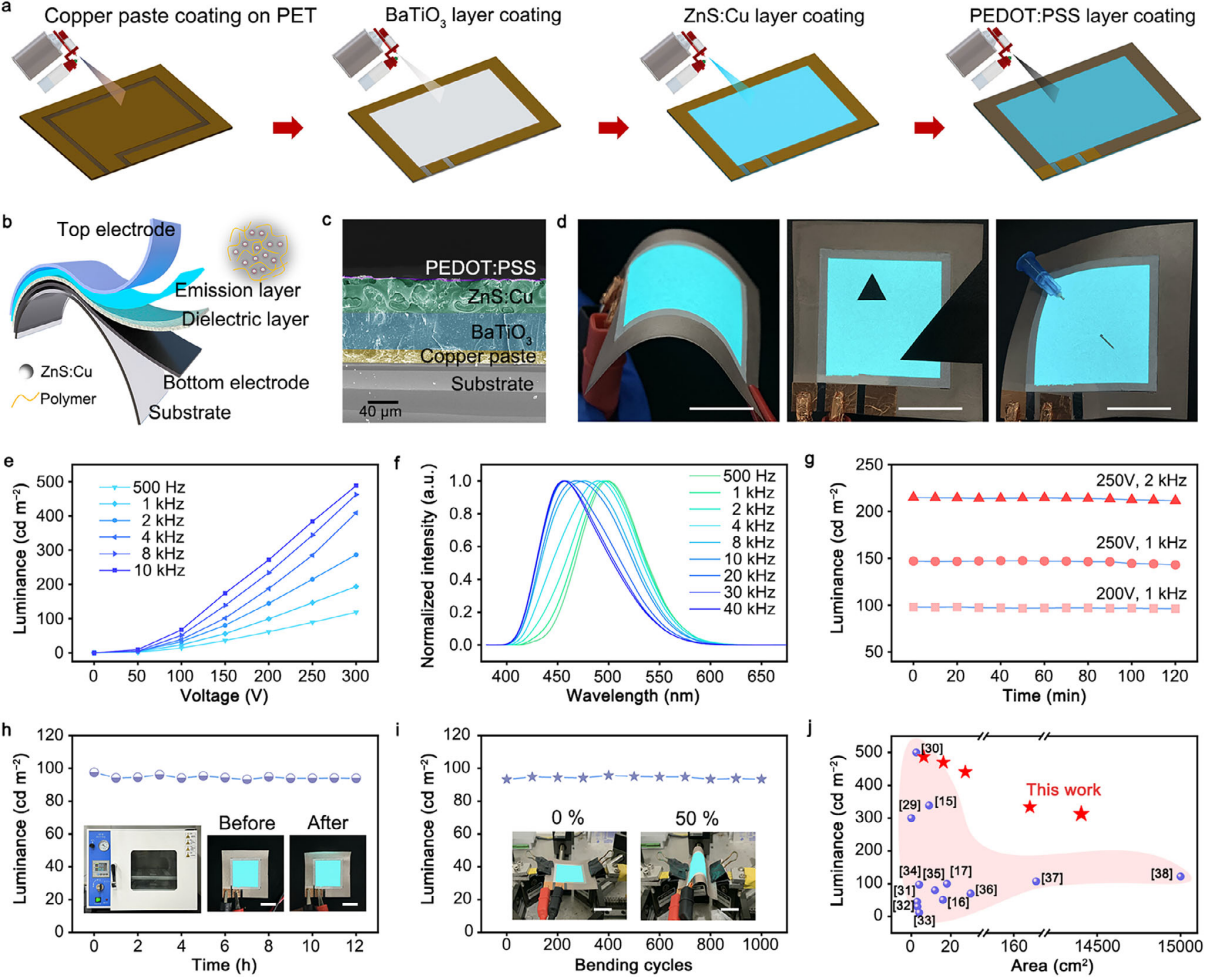
Electroluminescence performance of ACEL devices. (a) Schematics of aerosol spraying process used to fabricate ACEL devices. (b) Schematics of device structure. (c) SEM image of device's cross‐section structure. Scale bar is 40 µm. (d) Photographs of ACEL devices subjected to bending, cropping, and pinching tests. Scale bars are 2 cm. (e) Luminance as a function of applied voltage and frequency. (f) Emission peak shift with operating frequencies. (g) Electroluminescence stability test under various working conditions. (h) High‐temperature aging test. Insets show ACEL devices before and after heating at 85°C for 12 h. (i) Bending test. Insets display the device in its normal state and under 50% compressed. Scale bars are 2 cm. (j) Statistical comparison of area‐dependent luminance performance of electroluminescence devices.

Figure 3a shows the fully aerosol spraying process used to fabricate ACEL devices with AES‐modified luminescent ink. First, copper particle conductive ink was sprayed onto a flexible polyethylene terephthalate (PET) substrate with pre‐reserved insulating patterns. Next, BaTiO_3_ particle insulating ink was sprayed onto the conductive layer with exposed edge of bottom conductive layer. Importantly, our modified luminescent ink (ZnS:Cu/polymer) was then sprayed onto the insulating layer, following the same pattern as the insulating layer. Finally, a transparent top conductive layer of poly(3,4‐ethylenedioxythiophene)‐poly(styrene sulfonate) (PEDOT:PSS) was sprayed on to complete the device fabrication. The modified luminescent ink is also compatible with other fabrication processes, such as inkjet printing and knife coating, enabling the realization of large‐area full‐color and pixelated displays (Section S5). Figure 3b shows structural schematic of the ACEL device, with the layers from top to bottom as follows: a top electrode, an emission layer, a dielectric layer, a bottom electrode, and a flexible substrate. Figure 3c presents the corresponding scanning electron microscope (SEM) image, showing a cross‐section view with different functional layers highlighted by false colors (Section S6). The scale bar is 40 µm.

Due to the presence of the insulating layer, this ACEL device can only be lit up by applying an alternating current (AC), which can induce the accumulation and collision of positive and negative charges in the emission layer to achieve electroluminescence. Thanks to the design of thin film functional layer, the luminescent device exhibits excellent flexibility and bending performance, and localized damage does not affect the normal emission in other areas. Figure 3d shows that even after the device is bent at a large angle, cut, or punctured with a sharp object, the remaining intact areas can still maintain stable electroluminescence driven by AC.

Figure 3e shows the luminance variation as a function of applied AC voltage and frequency. After the applied voltage reaches the threshold voltage of 50 V, the device's luminance increases significantly in an approximately exponential manner with increasing voltage. Additionally, under the same voltage, when the AC frequency increases from 500 Hz to 10 kHz, the device's brightness increases multiplicatively. The experiments confirm that the device with 3 cm × 3 cm area achieves maximum luminance of nearly 500 cd m^−2^ at 300 V and 10 kHz. Interestingly, Figure 3f presents that the increase in current frequency can cause a shift in the emission peak, where the color changes from green (499 nm) to blue (456 nm) as the AC frequency increases from 500 Hz to 40 kHz, accompanied by a significant change in the CIE coordinates (Section S7). This blue shift at high frequency may be attributed to the excitation of higher energy levels created by the high‐energy carrier injection [23, 28]. However, under fixed voltage and frequency, the device's luminance can maintain long‐term stability. Figure 3g shows that under continuous operation at 220 V/1 kHz, 250 V/1 kHz, and 250 V/2 kHz for 2 h, the device's brightness remains almost unchanged from its initial value, with only a 1%–3% decrease in brightness after 2 h.

Additionally, the device stability was characterized under some extreme conditions such as high temperature, repeated folding and high humidity. When the as‐prepared ACEL device was continuously illuminated and placed in a dry oven at 85°C, its luminance was measured every hour. The experimental results indicate that the device exhibited excellent stability at high temperature, with its luminance dropping to only 96.5% of the initial value after 12 h (Figure 3h). Figure 3i shows that the device can achieve planar and large‐angle folding under mechanical clamping. After 1000 continuous folding cycles, the device's luminance showed almost no change, demonstrating its excellent flexibility. Moreover, the device exhibited outstanding luminescent stability after being immersed in water and subjected to horizontal stretching by the mechanical clamping (Section S8).

Our work addresses the issue of uniform particle dispersion of luminescent ink in the ACEL device, and this method can be extended for the fabrication of large‐scale ACEL devices. Using this fully aerosol spraying technique, we have successfully fabricated a series of luminescent devices with various luminescent areas, ranging from tens of square centimeters to several square meters (Section S9). Figure 3j provides a statistical comparison of the device emission area and corresponding luminance among electroluminescent devices based on luminescent particles or quantum dots [15, 16, 17, 29, 30, 31, 32, 33, 34, 35, 36, 37, 38]. More detailed performance comparisons can be found in Section S11. Our ACEL device achieved a maximum brightness of nearly 500 cd m^−2^ in small‐sized devices, setting a new benchmark for performance compared to previous reports. Notably, an ultra‐large device with an area of 1.44 m^2^ reached a brightness of 303.3 cd m^−2^, marking the highest brightness reported for meter‐scale ultra‐large ACEL devices. Compared to flexible OLED or perovskite devices, these ACEL devices offer more convenient manufacturing conditions and significantly lower production costs, showcasing unique advantages for large‐area backlight panels. Additionally, the stable characteristics of inorganic luminescent materials and the non‐current injection working mechanism confer excellent stability in extreme environments, including high temperatures, humidity, and ultraviolet radiation. This makes ACEL devices particularly suitable for use in harsh conditions such as outdoor, industrial, and military applications.

**FIGURE 4:**
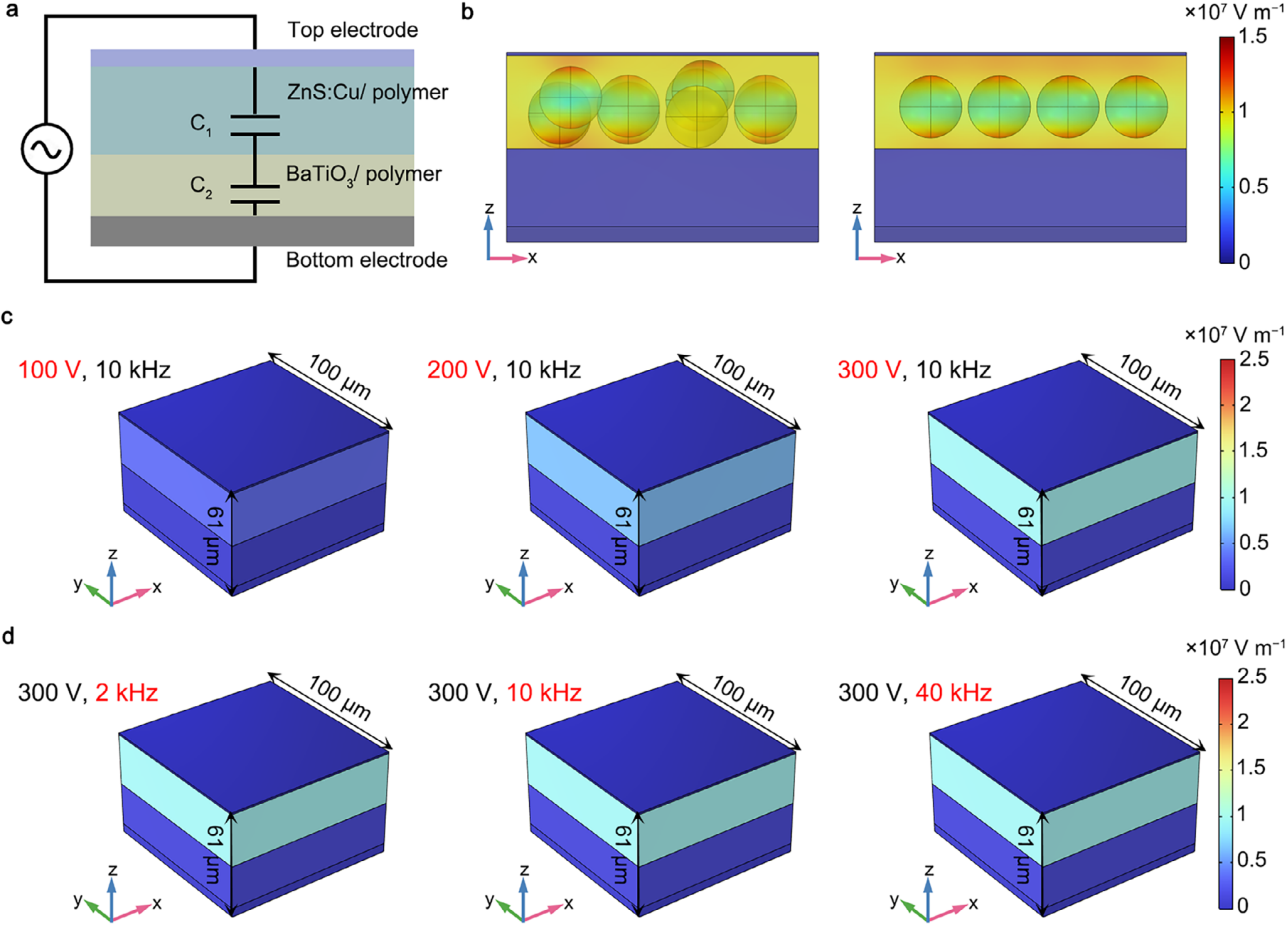
Simulations of device's electric field distribution for brightness enhancement. (a) The equivalent circuit of the ACEL device, showing two capacitors connected in series. (b) Simulations of electric field distribution on the cross‐section of ACEL devices with randomly arranged luminescent particles and uniformly distributed luminescent particles. (c) Simulations of drive‐voltage‐dependent electric field distribution. (d) Simulations of drive‐frequency‐dependent electric field distribution.

To better understand the working mechanism of the device and the reason for brightness enhancement, the ACEL device can be equivalent to two series‐connected capacitors, where capacitors *C*_1_ and *C*_2_ represents the layer capacitance of the ZnS:Cu/polymer luminescent layer and the BaTiO_3_/polymer dielectric layer, respectively (Figure 4a). To evaluate the circuit's performance, it is assumed that the luminescent layer and the dielectric layer are two homogeneous, isotropic, and linear dielectric materials. Under the influence of the electric field, the dielectric material is polarized into electric dipoles, which in turn generate bound charges. These bound charges produce an electric field opposite to the applied electric field. According to the relationship between the electric field (*E*) and the electric displacement vector (*D*), the electric field strengths of luminescent layer (*E*_1_) and dielectric layer (*E*_2_) are related to the dielectric constant (*ε*) of the materials as follows [15]:

(1) $$E_{1}=D_{1}\;/\varepsilon_{0}\;\varepsilon_{r1}=\delta /\varepsilon_{0}\varepsilon_{r1}$$

(2) $$E_{2}=D_{2}\;/\varepsilon_{0}\;\varepsilon_{r2}=\delta /\varepsilon_{0}\varepsilon_{r2}$$

where *D_1_* and *D_2_* is the electric displacement vector of luminescent layer and dielectric layer, respectively, which is an auxiliary vector introduced when discussing the relationship between charge distribution and electric field strength in the presence of a dielectric in an electrostatic field. ε_0_ is the vacuum dielectric constant, and ε_r_ is the dielectric constant of the functional material. Notably, the discussion is based on a cylindrical Gaussian surface, and according to Gauss's theorem for dielectric, *D*_1_ and *D*_2_ are equal to 𝛿, where 𝛿 is the free charge surface density on the pole plate.

Finite element simulations can further reveal the luminescent characteristics of the device. In COMSOL simulation software, by applying an AC to the device, the electric field distribution in the functional layers of the device can be simulated (Section S10). Figure 4b shows that the luminescent particles in the ink are simplified as small spheres in the modeling process, while the uncontrolled particle aggregation phenomenon in inks can be simulated using randomly distributed spheres. In contrast, the luminescent layer made from modified ink contains only regularly arranged spheres. Simulation results indicate that due to the dielectric constant difference between the luminescent and dielectric layers, the localized electric field is primarily concentrated in the luminescent layer, which has a lower dielectric constant. The enhanced electric field can increase the charge separation efficiency within the fluorescent particles, thereby improving the electroluminescent brightness of the device. Moreover, due to the different dielectric constants of fluorescent particles and the polymer matrix, stronger electric fields are mainly concentrated at top and bottom of the fluorescent particles. A disordered distribution of particles may cause a severely uneven electric field distribution within the luminescent layer, which is detrimental to the uniform expansion of current and uniform brightness in large‐area devices. These simulations indirectly confirm that the dispersion modification strategy of the luminescent particles has a significant effect on improving the large‐area uniformity of ACEL device.

Additionally, electromagnetic field simulations can be used to systematically investigate the effects of luminous area, driving voltage, and driving frequency on the electric field intensity distribution. Figure 4c shows that at a fixed driving frequency of 10 kHz, as the voltage of AC increases from 100 to 300 V, the electric field intensity in luminous layer increases from 0.5 × 10^7^ to 1.2 × 10^7^ V m^−1^. The enhanced electric field significantly increases the number of luminescent centers transitioning from the ground state to the excited state, thereby improving the material's electron–hole recombination probability and enhancing the device's electroluminescent intensity. However, under a fixed driving voltage of 300 V, Figure 4d shows that changing the driving frequency of AC does not alter the electric field intensity in the luminous layer. In contrast, experimental observations indicate that even with a constant voltage, altering the driving frequency still leads to a significant increase in brightness. This is because in actual devices, electric fields with higher frequencies can more effectively excite charge transitions and recombination in ZnS:Cu particles. Furthermore, from Equations (1) and (2), it can be concluded that the electric field intensity is independent of the device area. Therefore, it can be deduced that optimizing the dielectric properties of the luminescent layer and dielectric layer materials can achieve better brightness performance at lower driving voltages. For practical production applications, the light–emitting functionality of device can also be realized through a combination of series‐connected dry batteries (3–12 V) and AC transformers.

**FIGURE 5:**
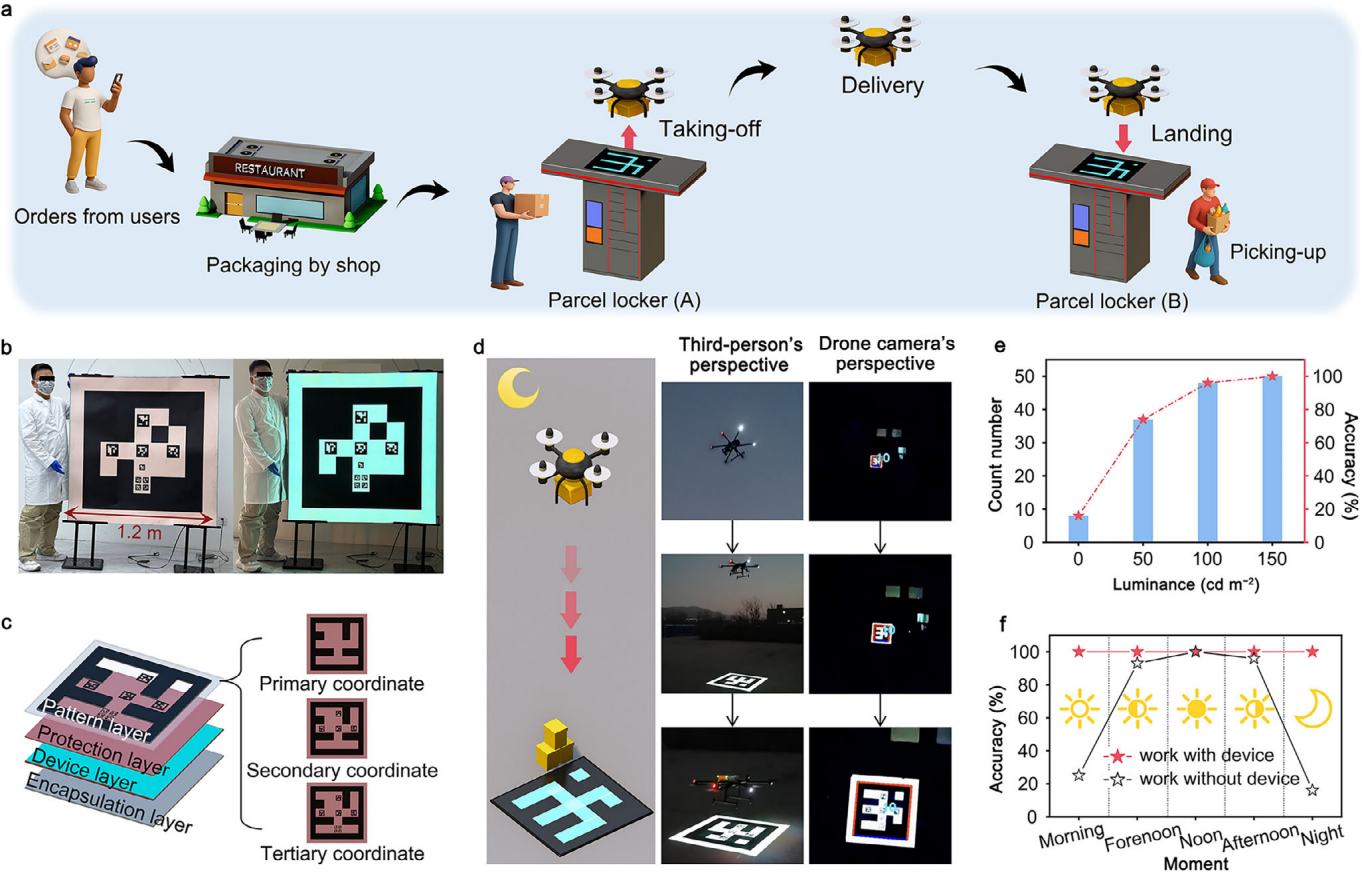
Meter‐scale ultra‐large‐area ACEL devices explored as navigation signs. (a) Schematics of the workflow for drone‐based logistics transportation of item delivery. (b) Photographs of the as‐fabricated ultra‐large ACEL device as glow‐in‐the‐dark navigation signs. (c) Schematics of device's functional layers with designed three‐level identification coordinates. (d) Schematics and photographs of the landing process for an industry drone working in dark. (e) The accuracy of image recognition by drones varies with different device luminance. (f) The recognition accuracy of drones with and without ACEL device changes throughout the day.

Recently, the new ecosystem of low‐altitude economy centered on drone logistics is rapidly developing, and thousands of drones will be able to take off and land simultaneously within a radius of 100 kilometers in the next five years. Some leading companies in China have already planned and explored a complete drone collaborative delivery system. Figure 5a illustrates the workflow for drone‐based logistics transportation of parcels and food delivery. After customers place an order via the app, the ordered items will be packed by shops and delivered to a parcel locker (A). The delivery drone then takes off from locker A, delivers items to locker B and lands autonomously. Finally, the customer retrieves the goods from locker B. This drone collaboration strategy will improve the transportation efficiency, save time and reduce labor cost.

However, there is currently no suitable technical solution for autonomous night‐time landing of drones using visual navigation. Specifically, there is a lack of navigation display technology that can present patterns via diffuse reflection during the day and actively emit light at night. Moreover, the light–emitting device must have a side length on the meter scale to accommodate large industrial drones performing navigation identification at altitudes of several hundred meters. Our fully‐aerosol spraying method provides an alternative approach for creating a new type of ultra‐large‐area display device that serves as a navigation sign for the autonomous takeoff and landing of industrial drones.

Figure 5b shows the 1.2 m × 1.2 m ultra‐thin flexible ACEL device, with a thickness of 0.5 mm and a luminous area of 1.2 m × 1.2 m. A multi‐level quick‐response (QR) code pattern has been designed and fabricated on the surface of the ACEL device to meet the navigation identification requirements for drones at different altitudes. Figure 5c presents the structural diagram of an ACEL device designed for potential commercial products primarily working in outdoors. This device is fabricated based on the previously described device layers (Figure 3b) and includes additional protective layers, QR code pattern layers, and a bottom encapsulation layer to ensure its practicality and stability. Inset images show the details of the QR code pattern design with three‐level identification coordinates.

The left pattern in Figure 5d illustrates the drone autonomously descending from high altitude to the ACEL navigation sign. The right patterns show photographs taken from both the third‐person's perspective and the drone camera's perspective. These photographs are extracted from the corresponding video footage (Section S12 and supporting Videos S1, S2). To further verify the reliability of the ACEL device, drones were flown to a 100‐meter altitude at night, where they completed autonomous visual landing under dark conditions. Fifty flight tests were conducted at varying light levels. The results revealed that when the device was not emitting light, the drone's successful landing accuracy was only 8%. As the brightness increased to 100 cd m^−2^ or above, the accuracy of landing approached 100% (Figure 5e). This demonstrates that sufficient electroluminescence brightness can provide excellent image contrast, meeting the visual autonomous landing requirements of drones in dark environments.

Additionally, Figure 5f shows the operation of the ACEL device in environments with sufficient daylight, including morning, noon, and afternoon, where the drone's autonomous landing accuracy reached 100%. In contrast, when using traditional black‐and‐white paper navigation signs, drones were unable to land correctly in environments with insufficient brightness, such as early mornings or evenings. This indicates that large‐area ACEL devices are expected to become an essential product for drone autonomous takeoff and landing navigation. In fact, the recognition accuracy of a drone's autonomous landing is contingent upon both the recognition capability of drone's camera and the contrast of display pattern on the ACEL device. In practical applications, complex weather conditions such as rain and fog can diminish visibility, thereby impacting the drone's ability to descend and execute autonomous landings. In the future, it is essential to further develop ACEL devices with linear or circular polarization emissions to ensure reliable performance in all weather conditions.

## 3 Conclusions

In summary, we proposed a scalable fully‐sprayed process to make meter‐scale ultra‐large flexible ACEL devices with planar stacked structures. By introducing AES surfactant to the synthesis of luminescent ink, the dispersibility and flowability of fluorescent particles in the luminescent ink have been improved. This surfactant engineering approach can be further extended to quantum dots, organic materials, and other luminescent ink systems, thereby enabling uniform coating of luminescent layer molecules and enhancing the brightness of luminescent devices. Our ACEL devices demonstrate excellent substrate compatibility and flexibility, with outstanding operational stability in high‐temperature, humid, and water‐rich environments. It also presents remarkable display performance with a brightness of nearly 500 cd m^−2^ in small‐sized devices and up to 303.3 cd m^−2^ in ultra‐large‐area devices exceeding meter‐scale size. Interestingly, these ACEL devices can be explored for application in the urban logistics delivery system of large industrial drones, serving as a luminous navigation sign that operates in all weather conditions for drone autonomous takeoff and landing, both during the day and night.

## 4 Methods

### 4.1 Materials

ZnS:Cu (99.99%, Sinopharm Chemical Reagent Co., Ltd., China), CuCl_2_·2H_2_O (99.99%, Aladdin), NH_4_Cl (99%, Aladdin), HCl (37%, Aladdin), Al(NO_3_)·9H_2_O (AR, Aladdin), HNO_3_ (AR, Sinopharm Chemical Reagent Co., Ltd., China), anhydrous ethanol (C_2_H_6_O, AR, Sinopharm Chemical Reagent Co., Ltd., China). Polyvinylpyrrolidone (PVP, 40 kDa, Aladdin), isophorone (97%, Aladdin), epoxy resin (Shenzhen Yilai Technology Co., Ltd., China), Poly(3,4‐ethylenedioxythiophene)‐poly(styrene sulfonate) (PEDOT:PSS, 5 wt.%, Aladdin), Dibasic ester (DBE, 99%, Aladdin), copper paste (Shenzhen Yilai Technology Co., Ltd., China), BaTiO_3_ ink (Shenzhen Yilai Technology Co., Ltd., China), N,N‐Dimethylformamide (DMF, 99.5%, Aladdin, AR), Sodium fatty alcohol polyoxyethylene ether sulphate (AES, 70%, Shanghai Yuanye Biotechnology Co., Ltd., China), Polyethylene terephthalate (PET, DuPont).

### 4.2 Preparation of Luminescent Ink

First, epoxy resin, DBE, and isophorone were weighed according to mass percentage proportions of 20%–25%, 40%–45%, and 25%–30%, respectively. The three raw materials were then mixed and stirred at 800 rpm for 1 h. Stirring was halted once the mixture became uniformly transparent, indicating the completion of the polymer solution preparation.The luminescent layer ink was prepared by dissolving ZnS:Cu particles, the polymer solution, and DMF in a mass ratio of 1:4:9.2. This mixture was stirred at 800 rpm for 2 h to ensure the uniform dispersion of the phosphor particles. For the modified ZnS:Cu luminescent inks, the surfactant AES was incorporated at concentrations of 0.5, 1, 1.5, and 2 wt.%, respectively. The resulting mixture was stirred at 800 rpm for 2 h to ensure complete dispersion of the AES. Finally, a series of highly dispersed luminescent layer inks with varying AES concentrations were successfully obtained.

### 4.3 Fabrication of ACEL Devices

First, a PVP layer was deposited onto a flexible PET substrate to improve the adhesion of subsequent functional layers. Copper paste was then uniformly applied via aerosol spraying onto the PVP‐modified PET, followed by drying at 60°C for 30 min to ensure complete solvent evaporation, forming the copper paste bottom electrode layer. Next, a BaTiO_3_/polymer solution was deposited using the same spraying method and dried at 60°C for 30 min to form the dielectric layer. The ZnS:Cu/polymer ink was then sprayed onto the dielectric layer and dried at 60°C for 50 min to form the luminescent layer. Finally, a PEDOT:PSS solution was sprayed as the transparent top electrode, and drying at 60°C for 30 min resulted in the ACEL devices. For ultra‐large‐area ACEL devices, a PET protective layer and pattern layer were further hot‐pressed encapsulated onto the top surface after fabrication. This is a standard process in the advertisement industry, wherein ACEL devices are strategically positioned between a top PET protective layer featuring a QR pattern and a bottom PET protective layer. Both layers possess an adhesive backing to facilitate easy attachment. To eliminate air bubbles, a roller is utilized to roll from the center toward the edges. Subsequently, a handheld heat‐sealing machine is employed to seal the edges of the luminescent pane. The sealing process is conducted at a temperature of 100°C and a pressure of 0.5 MPa. The PET protective film used is a transparent film with a thickness of 0.1 mm.

### 4.4 Electric Field Simulations

The 3D model of ACEL device was created using finite element software to simulate the electric field distribution in both the luminescent and dielectric layers. The layer thicknesses were set as follows: top electrode layer (1 µm), luminescent layer (30 µm), dielectric layer (25 µm), bottom electrode layer (5 µm). Other parameters of materials were set as follows: dielectric layer (conductivity 10^−9^ S m^−1^, dielectric constant 20), luminescent layer (conductivity 10^−9^ S m^−1^, dielectric constant 2). ZnS:Cu particles were modeled as spheres with a diameter of 20 µm.

### 4.5 Characterization

The luminance and colorimetry of ACEL devices were measured using a spectral color illuminance meter (OHSP‐350Z, Hangzhou Hongpu Optoelectronic Technology Co., Ltd., China). The AC power source consisted of a function generator (SDG1022X, Siglent, China) and a high‐voltage amplifier (HA‐820A, Pintech, China). XRD patterns were recorded on a diffractometer system (D8 Advance, Bruker) with Cu Kα source (λ = 1.5406 Å). SEM images and EDS analyses were conducted on an instrument (*IGMAHD, Zeiss, UK) at an acceleration voltage of 10 kV. FTIR spectra were recorded using a spectrometer (Nicolet iS 10, US). Low‐temperature PL spectra of the samples were collected with a confocal microscope system (WITec, alpha300) coupled to a liquid nitrogen cold trap. Transient decay curves were collected in a fluorescence spectrometer (FLS1000, Edinburgh Instruments). Ink contact angles were measured with a video optical contact angle meter (DSA100, KRUSS, Germany). The rheological properties of the ink were measured with a rotational rheometer (MCR 302e, Anton Paar). Fluorescence micrographs were captured with an industrial microscopy (Axio Imager 2, Zeiss, Germany). The high‐temperature resistance of the ACEL device was evaluated by a vacuum drying oven (DZF‐6020, China). The bending test was performed on a homemade test bench. The tensile test of the device was performed by a tensile testing machine (ZQ‐990LB, China). Film roughness was measured by a 3D optical microscope (LSM900, Zeiss, Germany).

## Conflicts of Interest

The authors declare no conflicts of interest.

## Supporting information




**Supporting File 1**: advs74003‐sup‐0001‐SuppMat.docx.

**Supporting File 2**: advs74003‐sup‐0002‐VideoS1.mp4.

**Supporting File 3**: advs74003‐sup‐0003‐VideoS2.mp4.

## Data Availability

The data that support the findings of this study are provided in the main text and the Supplementary Information. More data are available from the corresponding author upon request.
