# Supplementary material for: Meter‐Scale Ultra‐Large‐Area Flexible Electroluminescent Devices Enabled by Aerosol Spraying
Source: Adv Sci (Weinh). 2026 Jan 26;13(18):e23831. doi: 10.1002/advs.202523831 (PMC13042561; doi:10.1002/advs.202523831)
Supplement: Supplementary file 1 — Supporting File 1: advs74003‐sup‐0001‐SuppMat.docx. [file ADVS-13-e23831-s002.docx]

Supporting Information for

**Meter-Scale Ultra-Large-Area Flexible Electroluminescent Devices Enabled by** **Aerosol Spraying**

*Hao Song^1^, Liang Yi^1^, Lixiang Huang^1^, Wajid Ali^1^, Linhan Cai^1,2^, Chao Wang^1,2^, Jianhua Huang^1^, Ziwei Li^1,2^* and Anlian Pan^1, 3^**

^1^Hunan Institute of Optoelectronic Integration, College of Materials Science and Engineering, Hunan University, Changsha 410082, China.

^2^Changsha Blue Ink Technology Co., Ltd, Changsha 410012, China.

^3^School of Physics and Electronics, Hunan Normal University, Changsha 410081, China.

*E-mail: ziwei_li@hnu.edu.cn; anlian.pan@hnu.edu.cn

**S1. Characterization of ZnS:Cu powders.**

**S2. Viscosity test of luminescent layer inks.**

**S3. Effect of AES on the luminescent properties of ZnS:Cu.**

**S4. Surface morphology analysis of luminescent films related to AES concentration.**

**S5. Potential application of inkjet printing for fabricating RGB pixelated ACEL devices.**

**S6. SEM image of cross-sectional morphology of the device.**

**S7. Color variability of ACEL devices.**

**S8. Stability test of ACEL devices.**

**S9. Luminescent area dependent ACEL devices.**

**S10. Simulation of electric field distribution.**

**S11. Performance comparison of reported ACEL devices**

**S12. Supplementary videos**

**S1. Characterization of ZnS:Cu powders.**

The luminescent particles are prepared through a multi-step process involving doping, sintering, coating, and post-treatment. First, ZnS and CuCl_2_·2H_2_O were used as the main raw materials, with the doping concentration of Cu^2+^ controlled between 0.1-2 mol%. Taking the mass of ZnS as the basis, 5 wt% of NH_4_Cl was added as a flux. The raw material mixture was thoroughly ground in an agate mortar, dried at 80 °C for 2 hours, and then transferred to an alumina crucible. The crucible was placed in a tubular furnace and sintered at 1100 °C for 3 hours under a high-purity N_2_ atmosphere. After sintering, the furnace was naturally cooled to room temperature, and the sintered product was ground into a powder. Then, the powder was etched with 1 mol L^−1^ HCl for 30 minutes, followed by repeated washing with distilled water until the filtrate is neutral. The purified powder was dried at 80 °C and ground again to obtain the intermediate product (uncovered ZnS:Cu fluorescent particle).

Subsequently, a predetermined amount of the intermediate product was dispersed in an ethanol/water mixed solvent and sonicated for 30 minutes to form suspension A. Al(NO_3_)·9H_2_O was weighed based on a mass ratio of Al^3+^ to intermediate product of 1-10%, dissolved in ethanol, and magnetically stirred until a transparent solution B was formed. Solution B was slowly dripped into suspension A while adding HNO_3_ (0.1 mol L^−1^) to adjust the pH to 3-5. The mixture was then stirred at 60 °C for 2 hours, forming a stable Al(OH)_3_ sol coating on the surface of intermediate products. The coated particles were collected by centrifugation, washed 2-3 times with ethanol, and dried at 80 °C for 4 hours. Finally, the dried powder was calcined in a muffle furnace at 400 °C for 2 hours in ambient air to obtain the luminescent particles coated with an alumina layer, namely ZnS:Cu fluorescent particles.


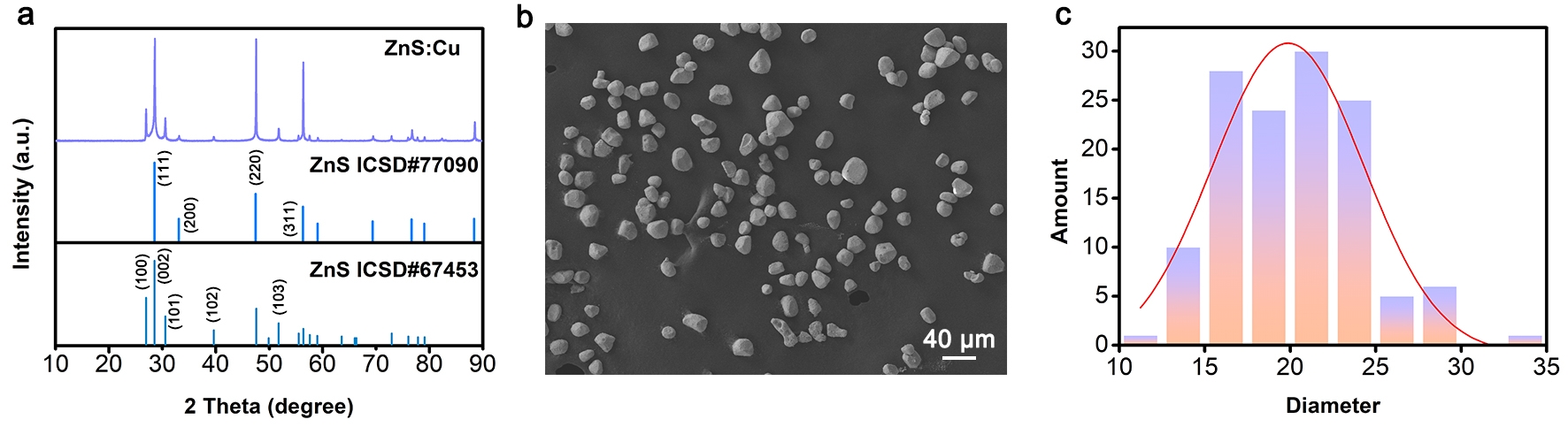


**Figure S1.** (a) XRD patterns of ZnS:Cu powders with standard reference patterns for cubic phase (ICSD#77090) and hexagonal (ICSD#67453) phase. The diffraction peaks of the ZnS:Cu sample at 2θ ≈ 28.5°, 47.5°, and 56.3° are well-indexed to the (111), (220), and (311) crystal planes of cubic ZnS (ICSD #77090). Meanwhile, additional diffraction peaks observed at 2θ ≈ 27.0°, 30.6°, and 39.6° can be assigned to the (100), (101), and (102) crystal planes of hexagonal ZnS (ICSD #67453). The coexistence of these phase-specific peaks confirms that the ZnS:Cu powder is a biphasic composite, with neither Cu⁺ substitution nor AlO_x_ coating affected its phase purity. (b) SEM image of ZnS:Cu powders. As presented in the SEM micrographs, the ZnS:Cu powders are composed of discrete particles with relatively uniform size, and these particles exhibit a smooth and defect-free surface. This favorable surface morphology is attributed to the uniform and dense AlO_x_ coating deposited on the ZnS:Cu particle surface. The AlO_x_ coating not only adheres tightly to the particle surface but also contributes to enhancing the environmental stability of the ZnS:Cu powder, which is crucial for maintaining the luminescent performance of ZnS:Cu during long-term storage or device operation. (c) Particle size distribution of ZnS:Cu powders. The average diameter of ZnS:Cu powders is measured to be 19.8 μm. Such a relatively small particle size facilitates the formation of highly dense and smooth luminescent layer films.


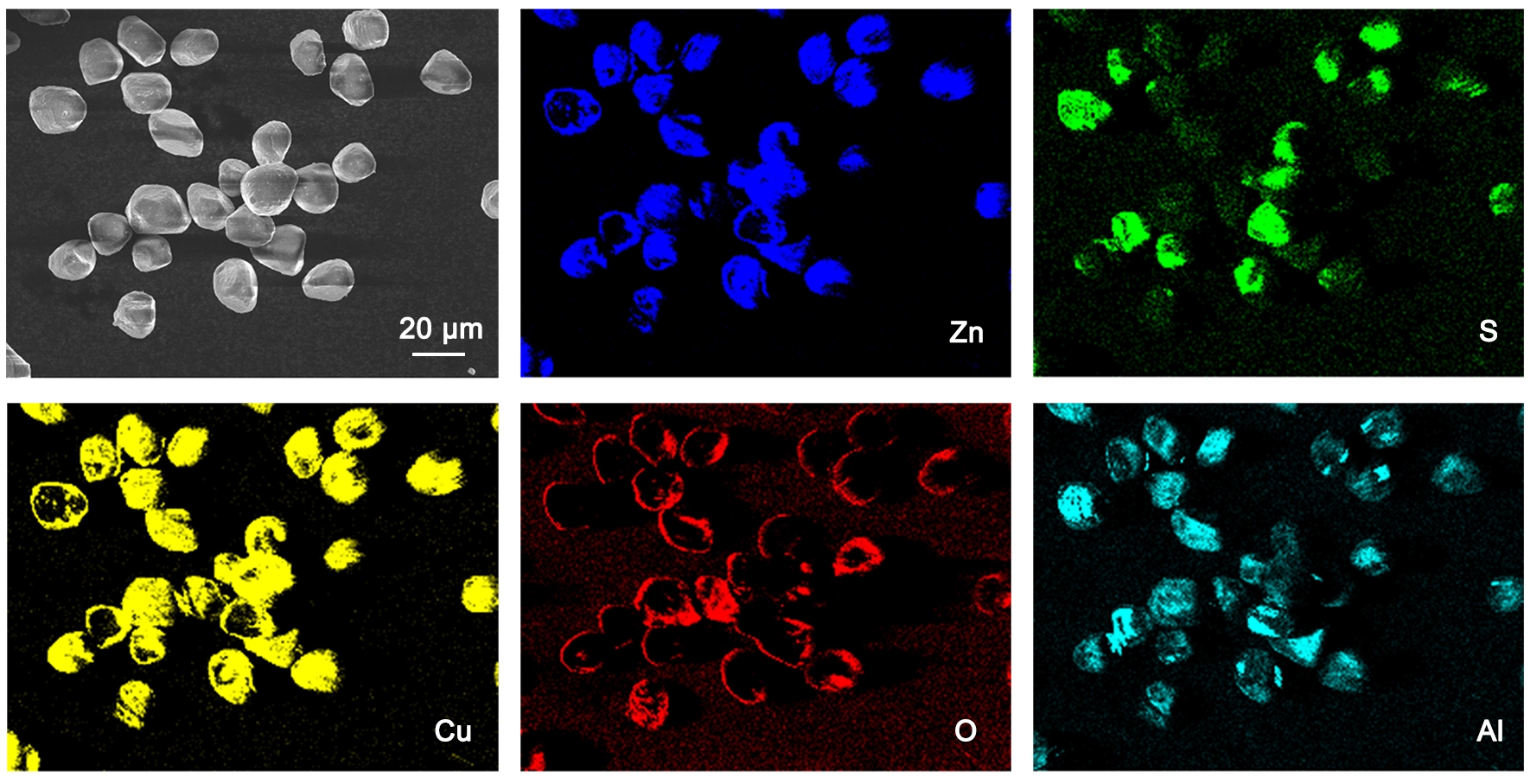


**Figure S2.** Elemental distribution mappings of ZnS:Cu powders. The EDX elemental distribution mappings clearly reveal the presence and spatial distribution of Zn, S, Cu, O, and Al elements within the ZnS:Cu particles.

**S2. Viscosity test of luminescent layer inks.**


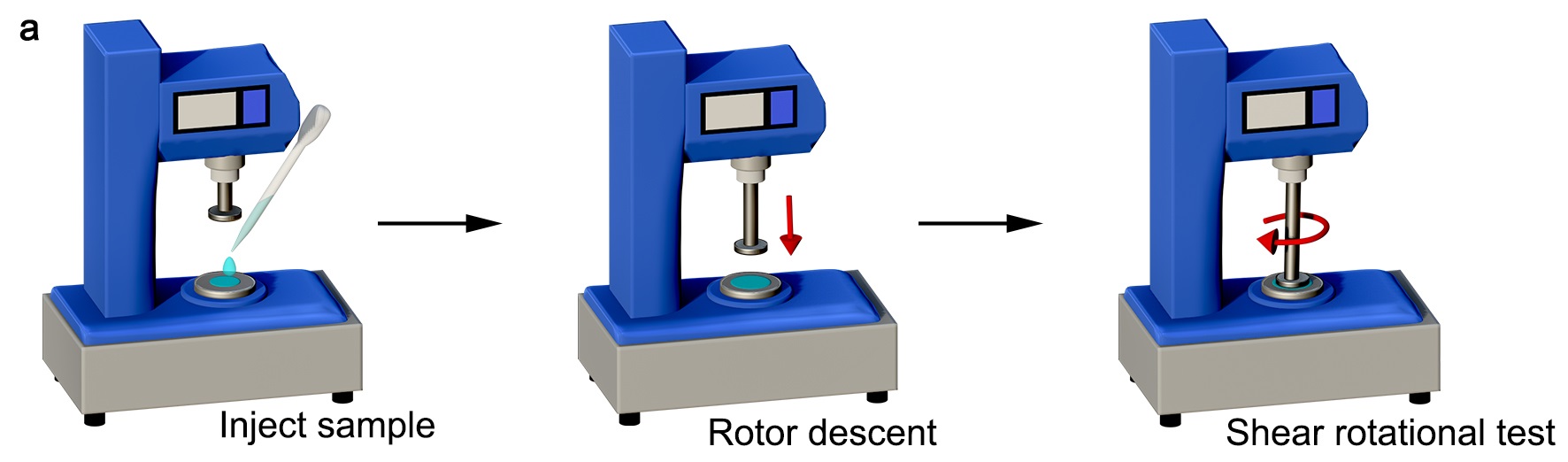


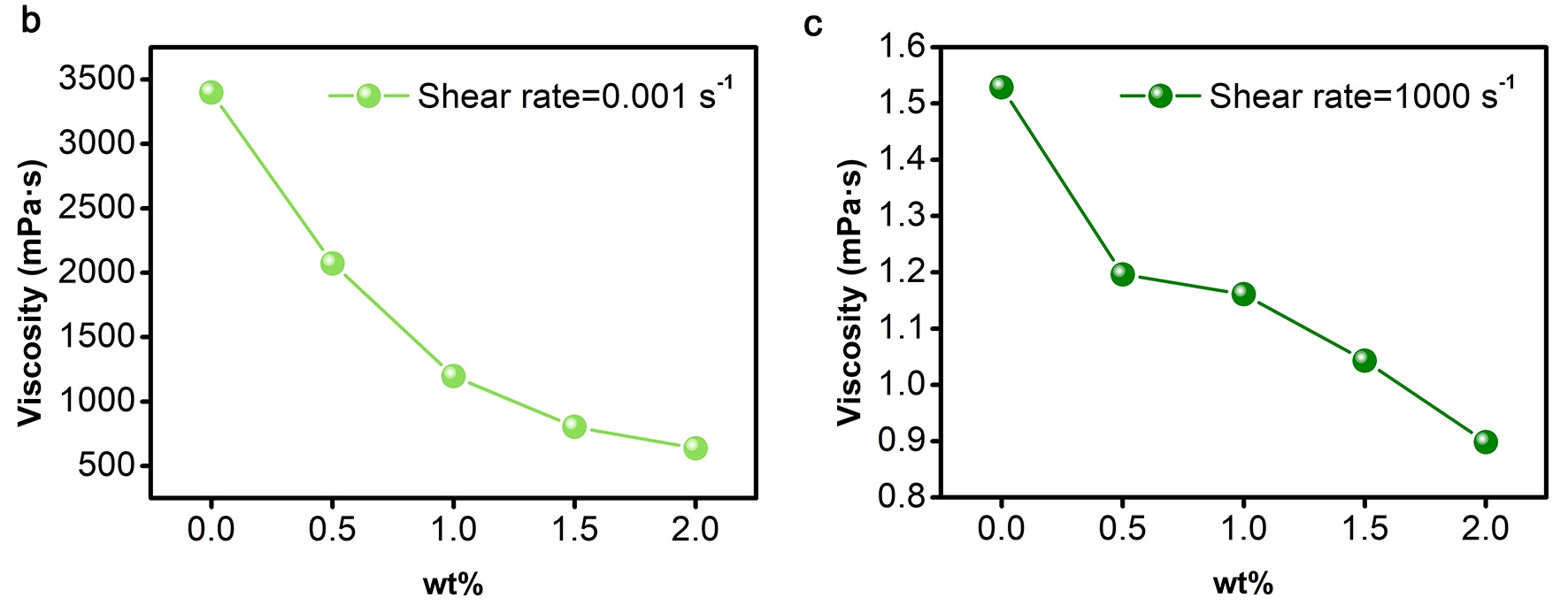


**Figure S3.** (a) Schematic illustration of the viscosity testing of the luminescent layer ink using a rotational rheometer. A preset volume of ink was injected onto the center of the lower stationary plate, ensuring full plate coverage without overflow. The upper rotor was slowly lowered to a predetermined gap from the lower plate, which was carefully calibrated to ensure uniform shear stress and minimize edge effects. Viscosity was measured under a preset shear rate, and the rheometer recorded real-time viscosity values during the test. (b) AES concentration-dependent ink viscosity at low shear rates. (c) AES concentration-dependent ink viscosity at high shear rates. The viscosity of the luminescent layer ink shows a clear concentration-dependent trend with respect to AES addition. Both at low and high shear rates, the ink modified with AES consistently exhibits lower viscosity than the unmodified control. This reduction in viscosity is attributed to the surfactant effect of AES, which reduces the interfacial tension between the ZnS:Cu particles and the ink matrix, mitigates particle-particle interactions, and facilitates the uniform dispersion of ZnS:Cu within the ink—consistent with the improved fluidity observed.

**S3. Effect of AES on the luminescent properties of ZnS:Cu.**


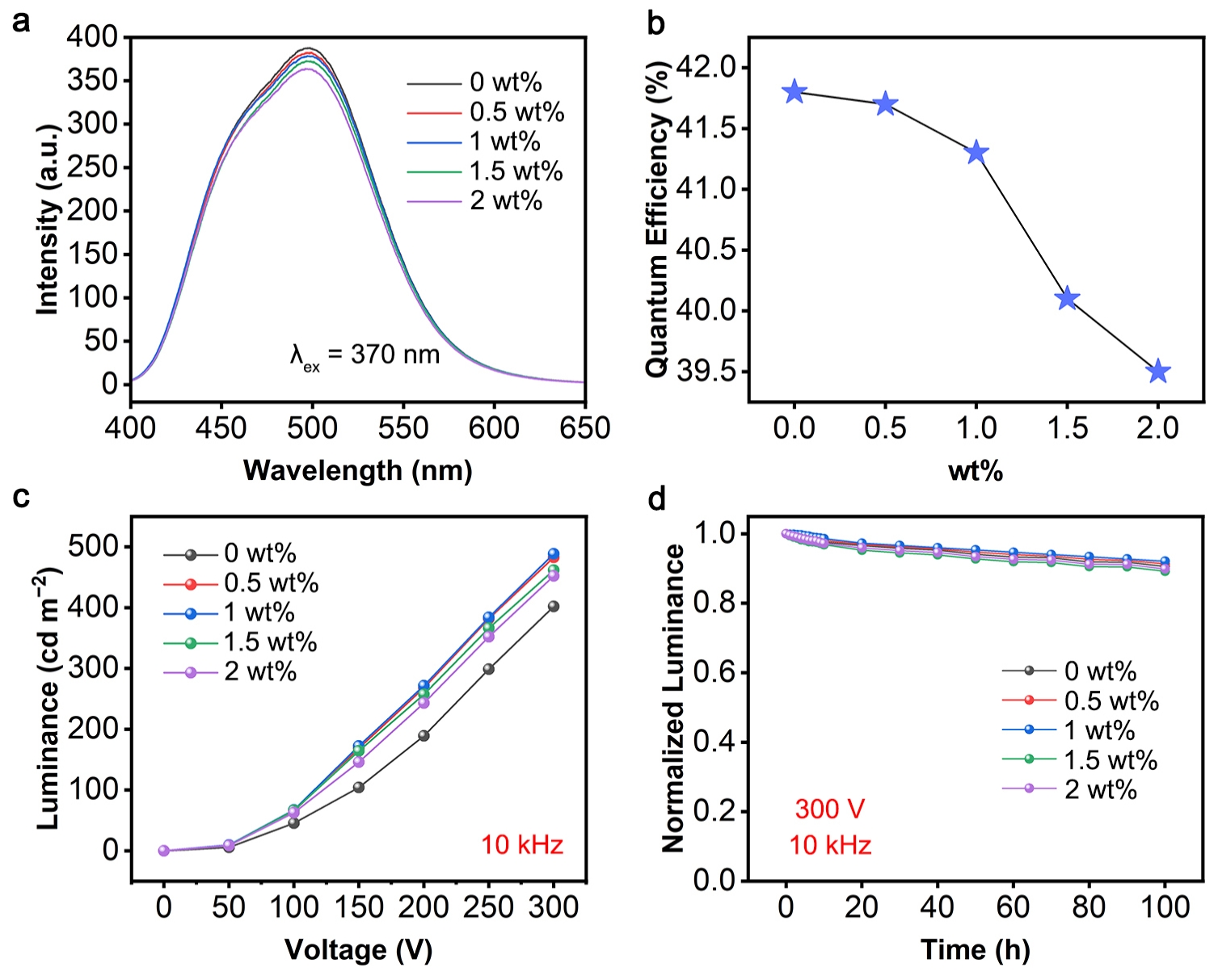


**Figure S4.** (a) PL spectra of ZnS:Cu powders modified with varying concentrations of AES. The spectral lines are nearly overlapping. (b) PLQY of ZnS:Cu powders modified with different AES concentrations, with values ranging from 39.5% to 41%, indicating no significant change. (c) Voltage-dependent luminance variations of ZnS:Cu powders modified with different AES concentrations. (d) Time-dependent decay of luminance, exhibiting a consistent attenuation trend.

**
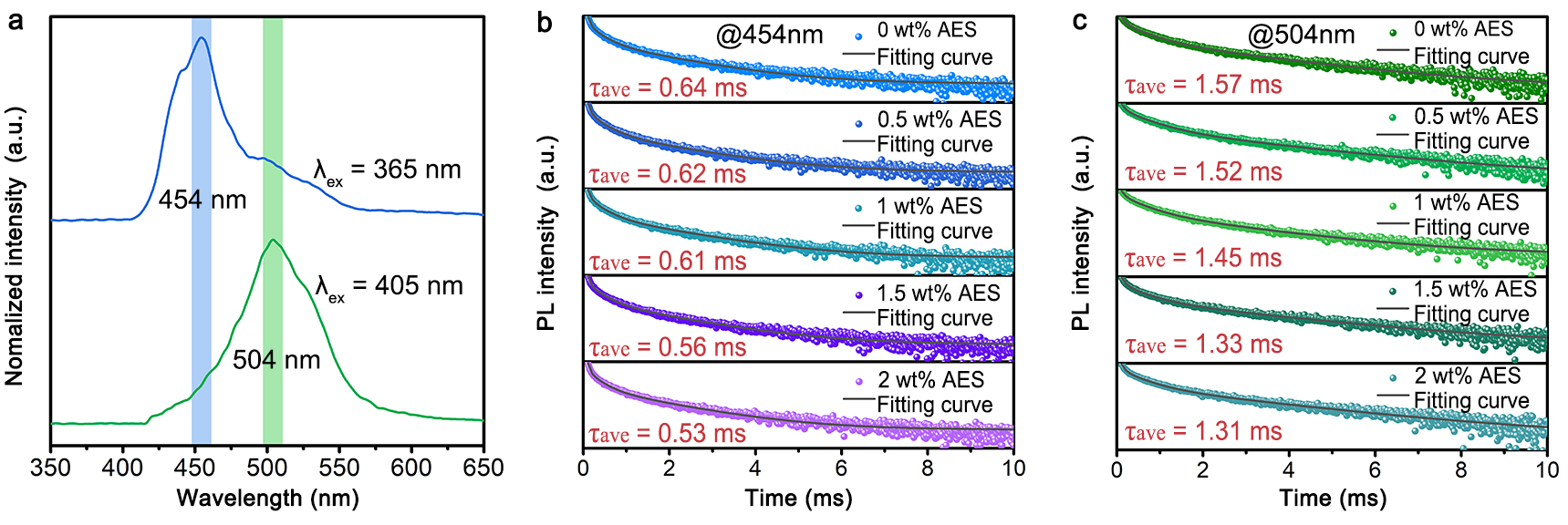
**

**Figure S5.** (a) Emission spectra of ZnS:Cu powders measured at 10 K and 10^−4^ Pa under the excitation wavelengths (*λ*_ex_) of 365 nm and 405 nm. Two prominent PL peaks are observed at 454 nm and 504 nm, which are attributed to the luminescent centers associated with interstitial Cu ions (Cu_i_) and substitutional Cu ions at Zn sites (Cu_Zn_), respectively^1, 2^. The ZnS:Cu powders exhibit distinct intensity ratios of these emission peaks when excited at 365 nm versus 405 nm. This phenomenon arises from the energy matching principle between excitation photons and luminescence centers, where specific excitation wavelengths selectively activate the two types of defect energy levels associated with Cu_i_ and Cu_Zn_. (b-c) PL decay curves of the ZnS:Cu powders modified with varying mass ratios of AES under 365 nm excitation. Specifically, the decay curves in S5b and S5c were recorded by monitoring the emission at 454 nm and 504 nm, respectively. The PL lifetime of ZnS:Cu powders can be estimated in a triple-exponential function, expressed as follow^3^:

$$I\left( t \right)= \sum_{i=1}^{3} A_{i}e^{\frac{t}{\tau_{i}}}= \sum A_{1}e^{\frac{t}{\tau_{1}}}+A_{1}e^{\frac{t}{\tau_{2}}}{+A}_{3}e^{\frac{t}{\tau_{3}}} (1)$$

where *I*(*t*) is the PL intensity, *t* is the time, *A_i_* represents the relative weights of the decay components at *t* = 0, *τ_i_* denotes the decay time for the corresponding exponential components. Actually, in this Cu-doped ZnS particle system, the associated excited state scenario is highly complex, involving factors such as bandgap radiative transitions, sulfide vacancies, and substitutional zinc. We cannot provide detailed deductions based on the limited detection results. But, the PL decay can be fitted using three components during the data fitting process: the first component (*A*_1_, *τ*_1_) is a fast decay representing rapid radiative recombination related to surface states or shallow defects; the second component (*A*_2_, *τ*_2_) corresponds to a moderate decay, reflecting the main radiative transitions with Cu^+^ as the activation center; the final component (*A*_3_, *τ*_3_) represents a slower decay associated with deep-level defects (such as sulfur vacancies, and interstitial zinc), which may involve delayed recombination or processes where carriers are trapped before being thermally released to luminescent centers.

Besides, the average lifetimes (*τ_ave_*) can be estimated in a function derived from the fitting parameters of the triple-exponential decay model, expressed as:

$$\tau_{ave}= \frac{A_{1}\tau_{1}^{2}+ A_{2}\tau_{2}^{2}+ A_{3}\tau_{3}^{2}}{A_{1}\tau_{1}+ A_{2}\tau_{2}+ A_{3}\tau_{3}} (2)$$

All the fitting parameters are listed in Table S1 and S2. For the untreated ZnS:Cu powders, the *τ_ave_* were determined to be 0.64 ms and 1.57 ms when monitoring emissions at 454 nm (blue light) and 504 nm (green light), respectively. When 1 wt% AES was added to modify the powder, no significant reduction in lifetime was observed. In contrast, as the AES content increased to 2 wt%, a notable shortening of lifetime of ZnS:Cu particles was detected. Based on these observations, 1 wt% was identified as the optimal AES addition amount for balancing the stability of particle’s luminescent lifetime and the potential processability improvements of the ink system.

**Table S1. Fitting data of TRPL decay curves (monitored at 454 nm)**

| Sample with AES | *A*_1_ | *τ*_1_ (ms) | *A*_2_ | *τ*_2_ (ms) | *A*_3_ | *τ*_3_ (ms) | *R*^2^ | *Τ*_ave_ (ms) |
| --- | --- | --- | --- | --- | --- | --- | --- | --- |
| 0 wt% | 0.13966 | 1.546 | 0.58654 | 0.30048 | 4.76485 | 0.04734 | 0.99845 | 0.64 |
| 0.5 wt% | 0.14146 | 1.52234 | 0.56171 | 0.30844 | 5.12837 | 0.04679 | 0.99836 | 0.62 |
| 1 wt% | 4.59501 | 0.04885 | 0.59342 | 0.28747 | 0.14639 | 1.45271 | 0.99824 | 0.61 |
| 1.5 wt% | 0.11372 | 1.52269 | 0.5297 | 0.31502 | 5.07062 | 0.04884 | 0.99869 | 0.56 |
| 2 wt% | 7.6083 | 0.03996 | 0.55081 | 0.2746 | 0.14232 | 1.43868 | 0.99848 | 0.53 |

**Table S2. Fitting data of TRPL decay curves (monitored at 504 nm)**

| Sample with AES | *A*_1_ | *τ*_1_ (ms) | *A*_2_ | *τ*_2_ (ms) | *A*_3_ | *τ*_3_ (ms) | *R*^2^ | *Τ*_ave_ (ms) |
| --- | --- | --- | --- | --- | --- | --- | --- | --- |
| 0 wt% | 0.5483 | 0.48797 | 0.1783 | 2.6155 | 1.74112 | 0.0754 | 0.99781 | 1.57 |
| 0.5 wt% | 7.6083 | 0.03996 | 0.55081 | 0.2746 | 0.14232 | 1.43868 | 0.99785 | 1.52 |
| 1 wt% | 0.16253 | 2.54281 | 0.60405 | 0.56064 | 1.23815 | 0.08989 | 0.99823 | 1.45 |
| 1.5 wt% | 1.21954 | 0.09017 | 0.60353 | 0.51185 | 0.18038 | 2.27936 | 0.99847 | 1.33 |
| 2 wt% | 0.19733 | 2.17729 | 0.61621 | 0.4945 | 1.19864 | 0.08415 | 0.99847 | 1.31 |

**S4.** **Surface morphology** **analysis of luminescent films** **related to AES concentration.**


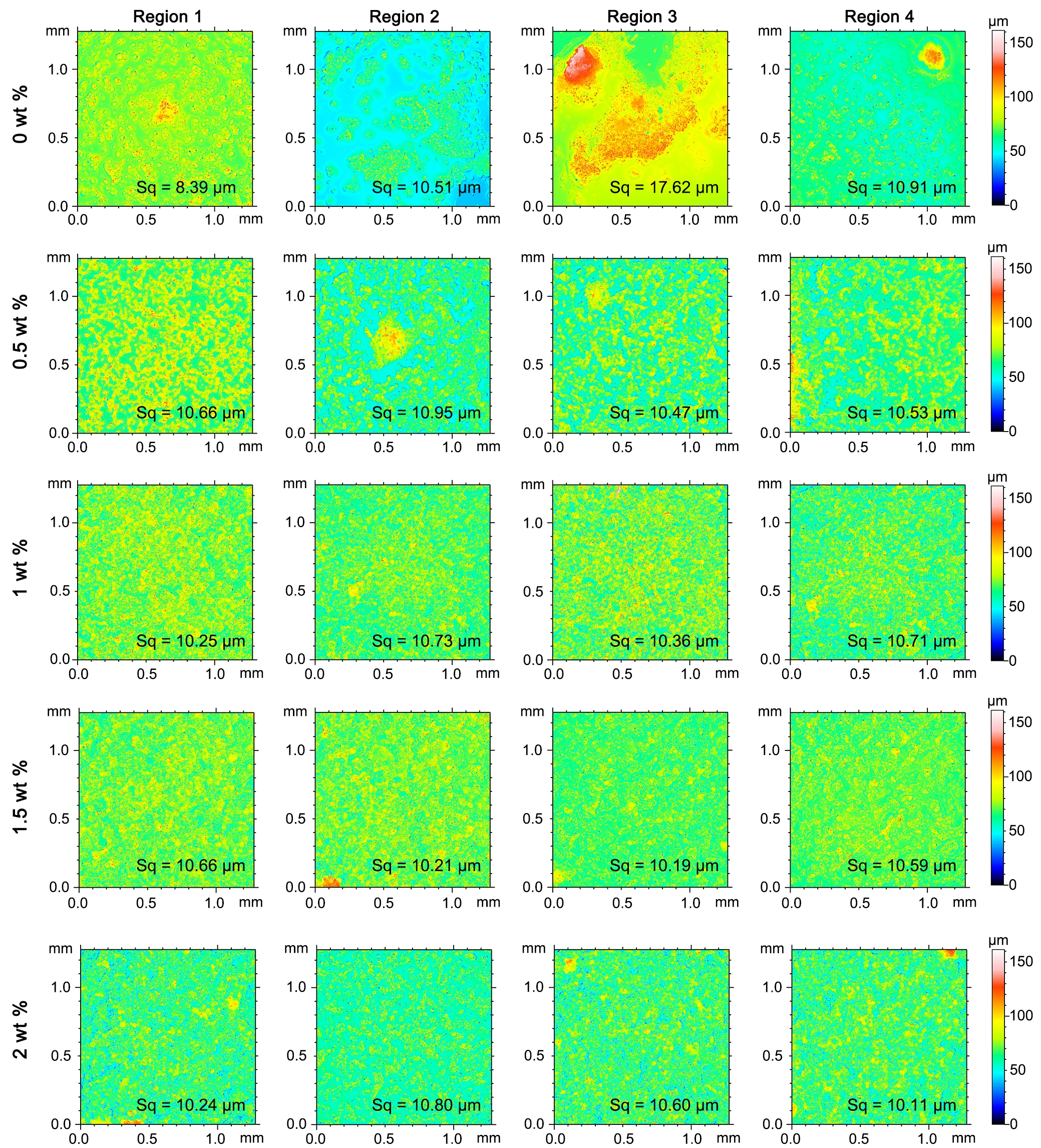


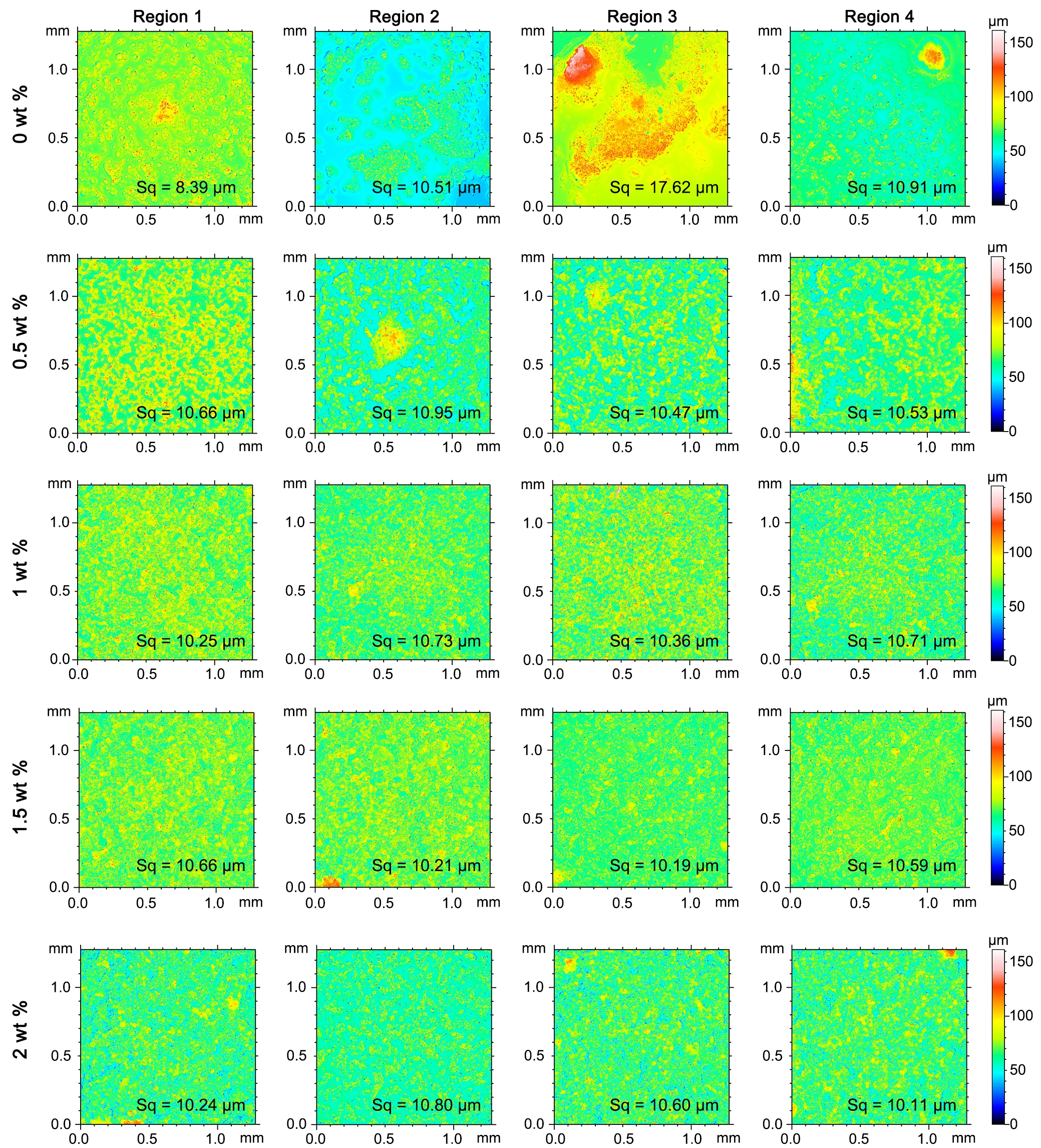


**Figure S6.** Surface morphology analysis of luminescent films modified with various concentrations of AES ranging from 0 to 2 wt%. Both unmodified ZnS:Cu ink and AES-modified ZnS:Cu inks were sprayed onto a dielectric/conductive layer/PET device substrate and dried at 60 °C for 15 minutes to prepare semi-finished luminescent devices. The prepared device samples were then examined using a 3D optical microscope to analyze the surface morphology of the micrometer-thick films and obtain qualitative roughness data (denoted as Sq). To assess the reproducibility of the measurements, four random positions on each sample were characterized. The 2D morphology images of unmodified luminescent films displayed significant unevenness, which can be attributed to the uncontrolled agglomeration of ZnS:Cu particles. The roughness of this sample was observed to fluctuate widely, ranging from 8.39 to 17.62 μm. In contrast, samples modified with different concentrations of AES exhibited significantly improved uniformity in film roughness, with surface roughness across different positions maintaining approximately 10 μm. These results on surface morphology convincingly demonstrate that AES molecules significantly improve particle dispersion, which will facilitate the fabrication of meter-scale ultra-large-area flexible electroluminescent devices through the aerosol spraying process. The morphology characterization results are consistent with the brightness uniformity of the light-emitting device compared to Figure 1 in the main text.

**S5. Potential application of inkjet printing for fabricating RGB pixelated ACEL devices.**


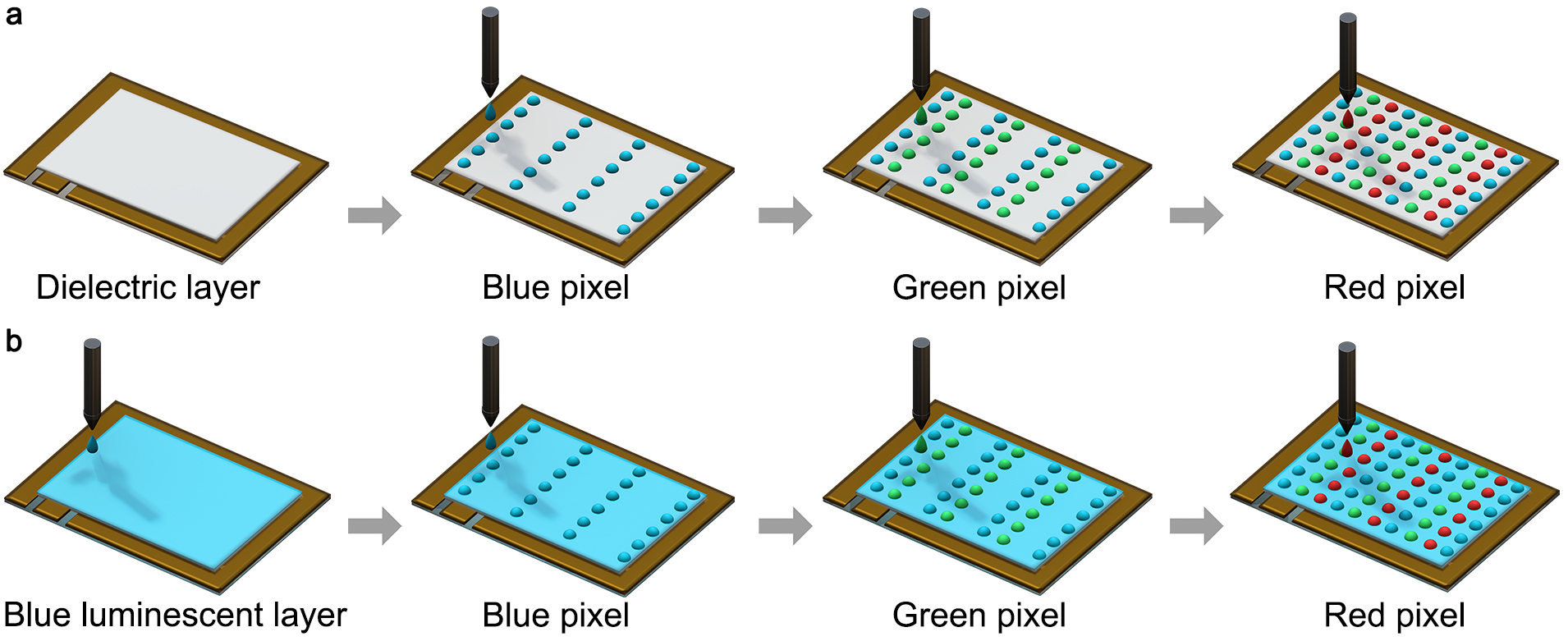


**Figure S7.** (a) Schematic of inkjet printing of inorganic luminescent ink for fabricating RGB pixelated ACEL devices. (b) Schematic of inkjet printing of large-scale backlight luminescent ink and RGB pixelated color-conversion layers for full-color ACEL devices.

This modified luminescent ink is compatible with inkjet printing technology for the fabrication of large-sized luminescent devices and RGB pixelated full-color displays. Figure S7a presents a schematic of inkjet printing RGB pixels on a dielectric/conductive substrate to create large-area full-color luminescent devices. Blue, green, and red luminescent inks are sequentially arrayed and inkjet printed onto the substrate, followed by the deposition of a top conductive layer to complete the device fabrication. To achieve dynamic active drive display capabilities, careful consideration must be given to the lead design of the top driving circuit. Additionally, Figure S7b illustrates a full-color device designed for pixelated displays, which integrates a backlighting layer with color-conversion layers. Initially, blue or white emissive ink is uniformly deposited over a large area onto the dielectric/conductive substrate via inkjet printing to serve as the blue or white backlight. Subsequently, an RGB pixel array is printed to obtain the color conversion luminescent layer. Lastly, careful attention must be paid to the design and fabrication process of the liquid crystal layer and the active drive layer at the top. Subsequently, an RGB pixel array is printed to obtain the color conversion luminescent layer. Lastly, careful attention must be paid to the design and fabrication process of the liquid crystal layer and the active drive layer at the top.

Due to the size limitations of the luminescent particles, this ink cannot be utilized with inkjet printheads that have small nozzle diameters, resulting in a pixel density and resolution that cannot match those of OLED devices. However, it still offers certain advantages for large-sized pixel displays in extreme weather conditions and outdoor scenarios. Relevant research teams have demonstrated the feasibility of using such materials in inkjet printing to fabricate patterned luminescent devices.

**S6.** **SEM image of cross-sectional morphology of the device.**

**
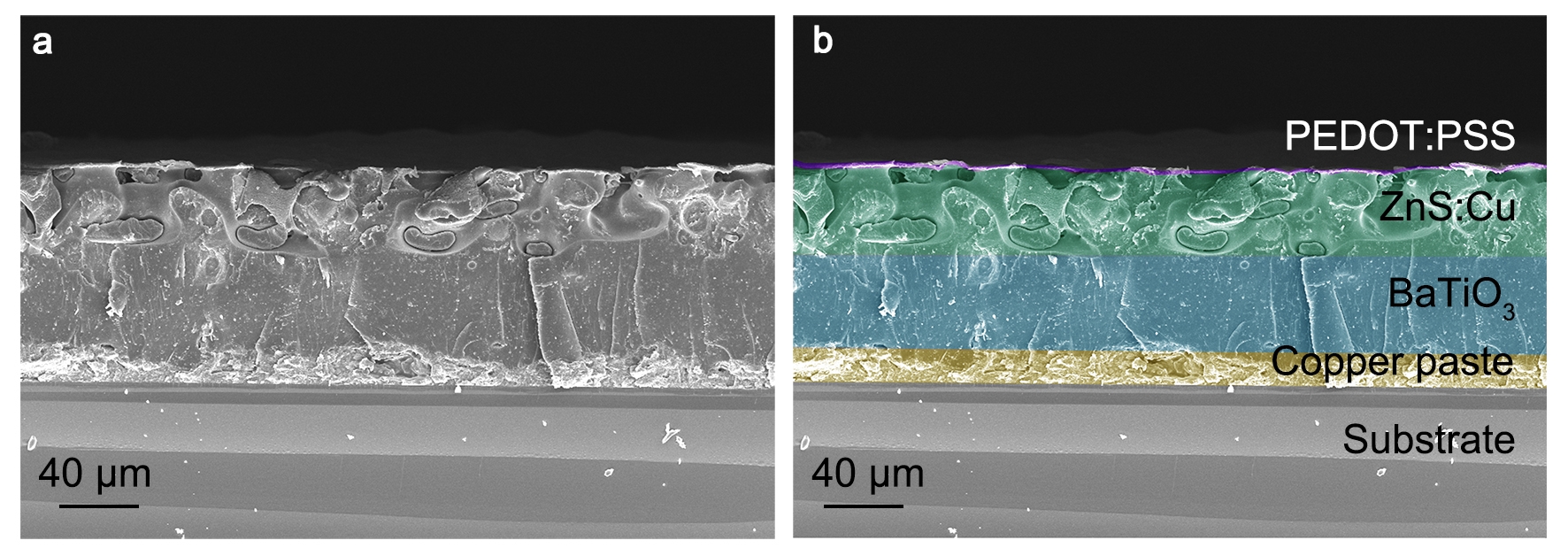
**

**Figure S8.** (a) Cross-sectional SEM image of the ACEL device. (b) Corresponding pseudo-colored image. From top to bottom, the ACEL device consists of a top electrode layer, a luminescent layer, a dielectric layer, a bottom electrode and substrate. Notably, all functional layers of the device exhibit excellent uniformity and planarity. The shared polymer matrix utilized in both the luminescent and dielectric layers ensures seamless integration between these two layers, thereby minimizing the risk of discontinuities or inhomogeneities in the electric field distribution across the device.

**S7. Color variability of ACEL devices.**

**
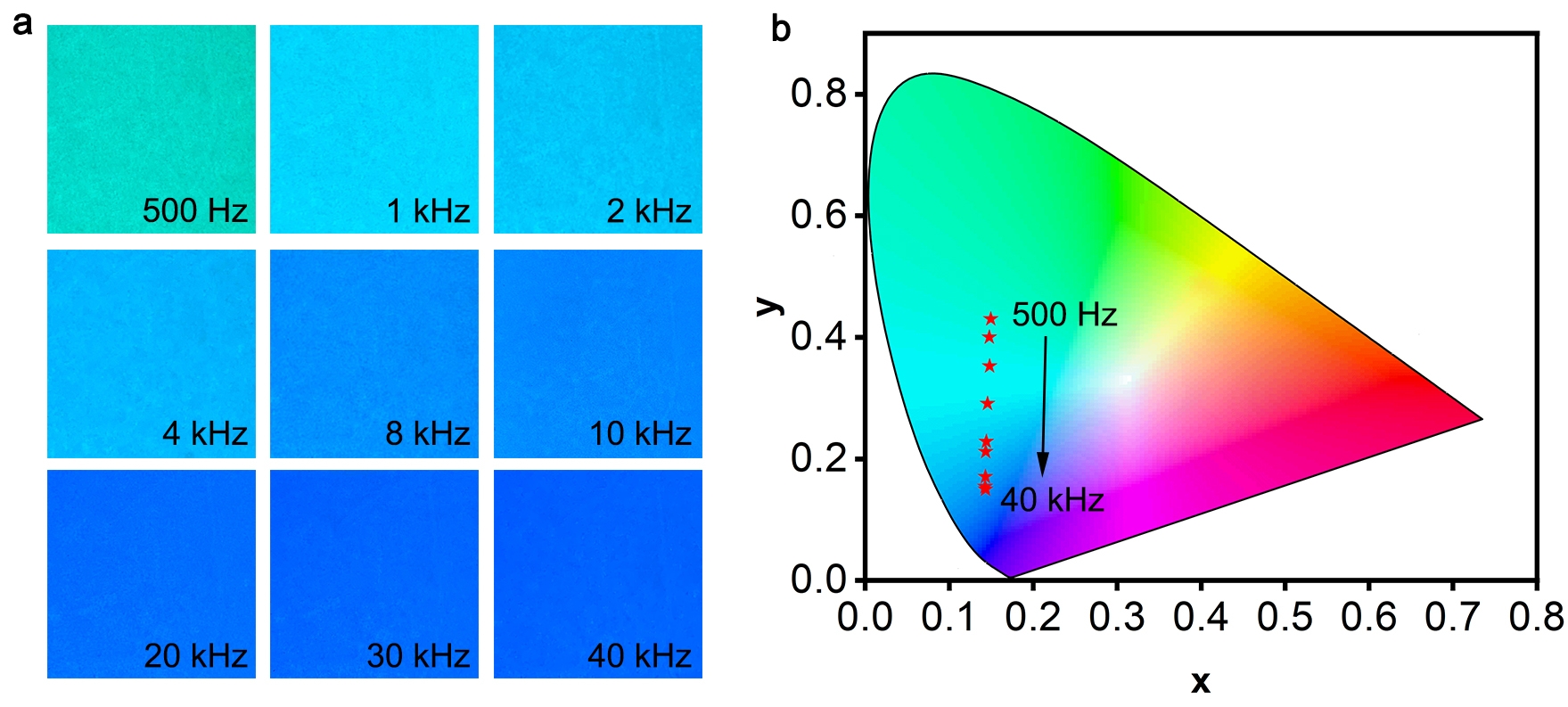
**

**Figure S9.** (a) Drive frequency dependent of ACEL device photographs under a fixed AC voltage of 300 V. (b) The CIE 1931 chromaticity coordinates of the device under various driving frequencies. Notably, as the driving frequency increased from 500 Hz to 40 kHz, the CIE 1931 coordinates of the device exhibited a gradual blue shift from (0.1494, 0.4302) to (0.1432, 0.1497). This shift toward shorter wavelengths (blue region) at high frequencies is a typical frequency-dependent phenomenon in ACEL devices, which can be attributed to the enhanced injection and transport of high-energy carriers (e.g., hot electrons) under elevated frequency conditions^4^. These high-energy carriers preferentially excite the higher-energy excited states of the luminescent centers (the high-lying excited states of interstitial Cu ions (Cu_i_) in ZnS:Cu phosphors)^1^. This frequency-tunable color behavior also indicates that the device enables fine regulation of luminescent color without altering the material composition, which is advantageous for flexible display applications requiring dynamic color adjustment.

**S8. Stability test of ACEL devices.**


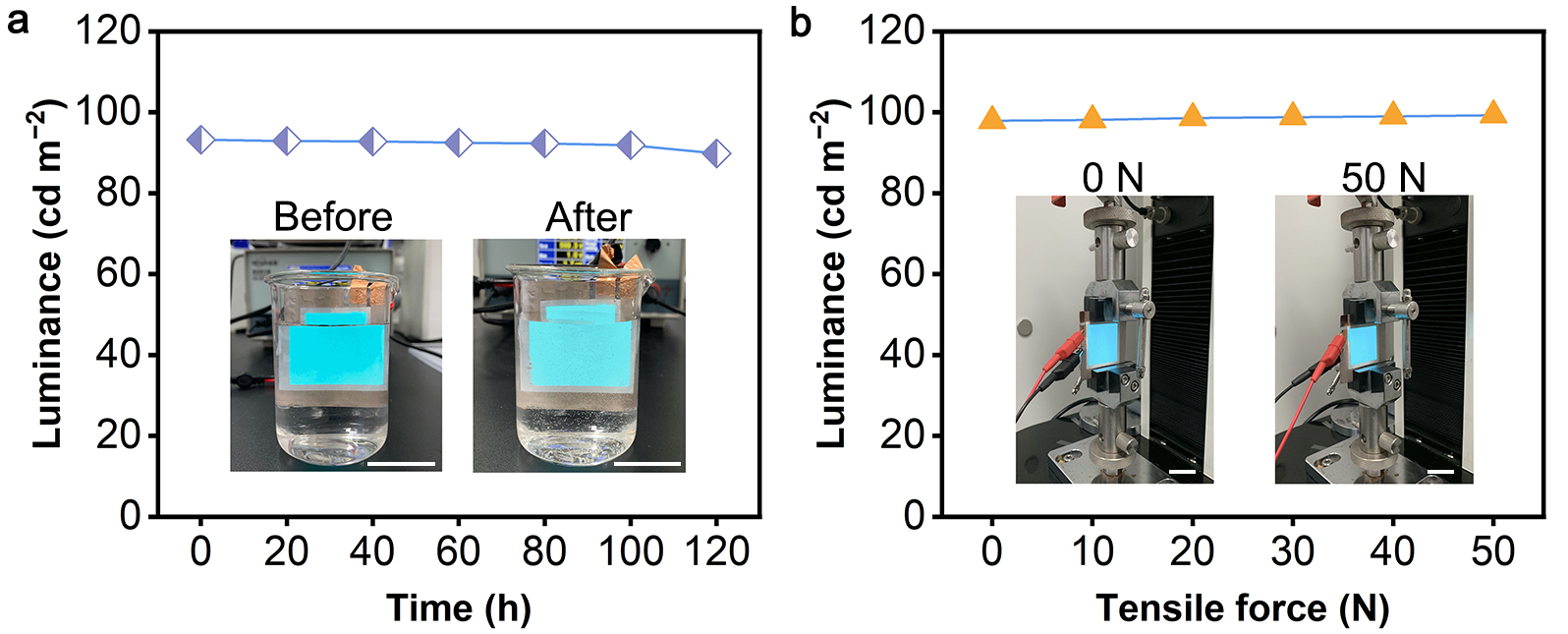


**Figure S10.** (a) Waterproof Test. Time-dependent luminance variations of a small-size ACEL device (3 cm × 3 cm) immersed in water for 2 hours are presented. Before the test, the as-prepared device was encapsulated with PDMS sealing layers. During a continuous monitoring period of 120 minutes, the luminance decreased by only 3.6%. Insets provide photographs of the testing setup. Scale bars: 2 cm. (b) Tensile Test. Luminance variations of the small-size ACEL device under tensile stress ranging from 0 N to 50 N are shown. Throughout the test, the luminance of the device remained nearly unchanged, demonstrating robust structural integrity and stable electrical contact even under mechanical strain. Insets display photographs of the testing procedure. Scale bars: 2 cm.


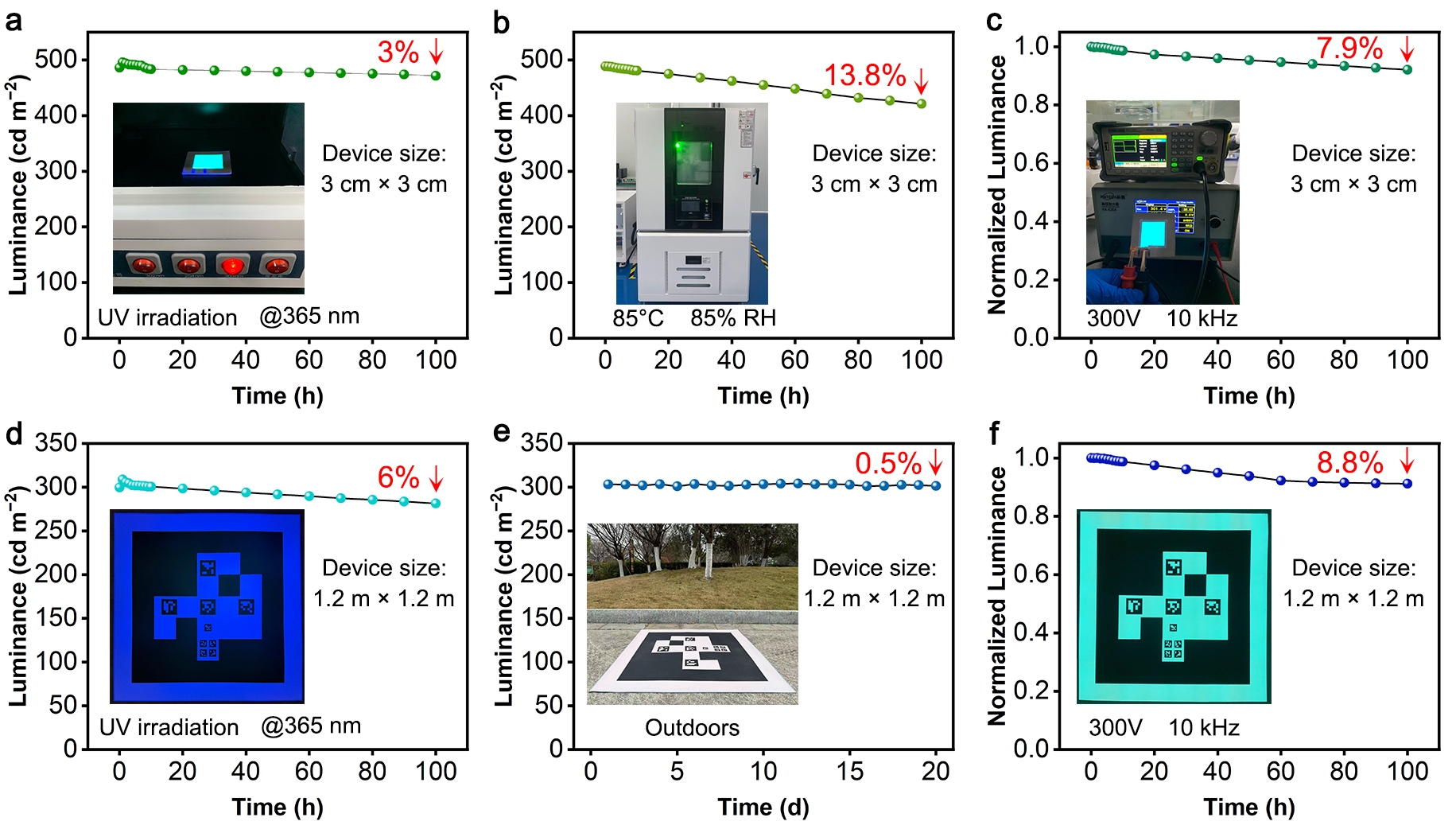


**Figure S11.** Time-dependent luminance variations of small-size ACEL device (3 cm × 3 cm) exposed by UV light (a), placed in a constant temperature and humidity chamber (b), and subjected to a continuous aging test at room temperature (c) Time-dependent luminance variations of large-size ACEL device (1.2 m × 1.2 cm) exposed to UV light (d), positioned outdoors under weather exposure (e), and undergoing a continuous aging test at room temperature (f).

Figure S11a illustrates the time-dependent luminance variations of small-sized devices exposed to UV light. During the experiment, the device was placed in an environment subjected to ultraviolet irradiation, and after a specified duration, it was powered on to test brightness. Initially, a slight enhancement in luminance was observed when the device was first exposed to UV radiation. This phenomenon can be attributed to the trapping of carriers within shallow defect energy levels in the phosphor materials as a result of UV irradiation. When an alternating electric field is applied, these trapped carriers undergo radiative transitions, contributing to the observed enhancement in the device’s luminance. After 100 hours of prolonged UV exposure, the brightness of both small and large devices decreased by 3%. Figure S11b demonstrates that the luminance of small-sized devices decreased by 13.8% when stored in a constant temperature and humidity chamber, indicating a reduction in brightness over the specified interval. Furthermore, Figure S11c shows a luminance decrease of 7.9% in an atmospheric environment following 100 hours of continuous operation. Similarly, for the large-scale devices, the luminance decreased by 6% under UV exposure, by 0.5% when exposed to outdoor conditions, and by 8.8% during continuous operation. The weather information during the 20 days’ test is listed in Table S3.

**Table S3. List of outdoor environmental parameters over 20 days.**

| Days | Maximum temperature | Minimum temperature | Average air humidity | Days | Maximum temperature | Minimum temperature | Average air humidity |
| --- | --- | --- | --- | --- | --- | --- | --- |
| 1 | 17 °C | 5 °C | 65 % | 11 | 14 °C | 3 °C | 60 % |
| 2 | 18 °C | 9 °C | 60 % | 12 | 17 °C | 3 °C | 55 % |
| 3 | 23 °C | 12 °C | 55 % | 13 | 18 °C | 6 °C | 65 % |
| 4 | 16 °C | 7 °C | 85 % | 14 | 16 °C | 8 °C | 80 % |
| 5 | 12 °C | 6 °C | 70 % | 15 | 14 °C | 7 °C | 80 % |
| 6 | 17 °C | 8 °C | 65 % | 16 | 10 °C | 6 °C | 80 % |
| 7 | 10 °C | 7 °C | 80 % | 17 | 10 °C | 6 °C | 75 % |
| 8 | 10 °C | 7 °C | 80 % | 18 | 13 °C | 7 °C | 70 % |
| 9 | 13 °C | 4 °C | 75 % | 19 | 13 °C | 7 °C | 70 % |
| 10 | 11 °C | 2 °C | 65 % | 20 | 16 °C | 7 °C | 75 % |

**S9. Luminescent area dependent ACEL devices.**


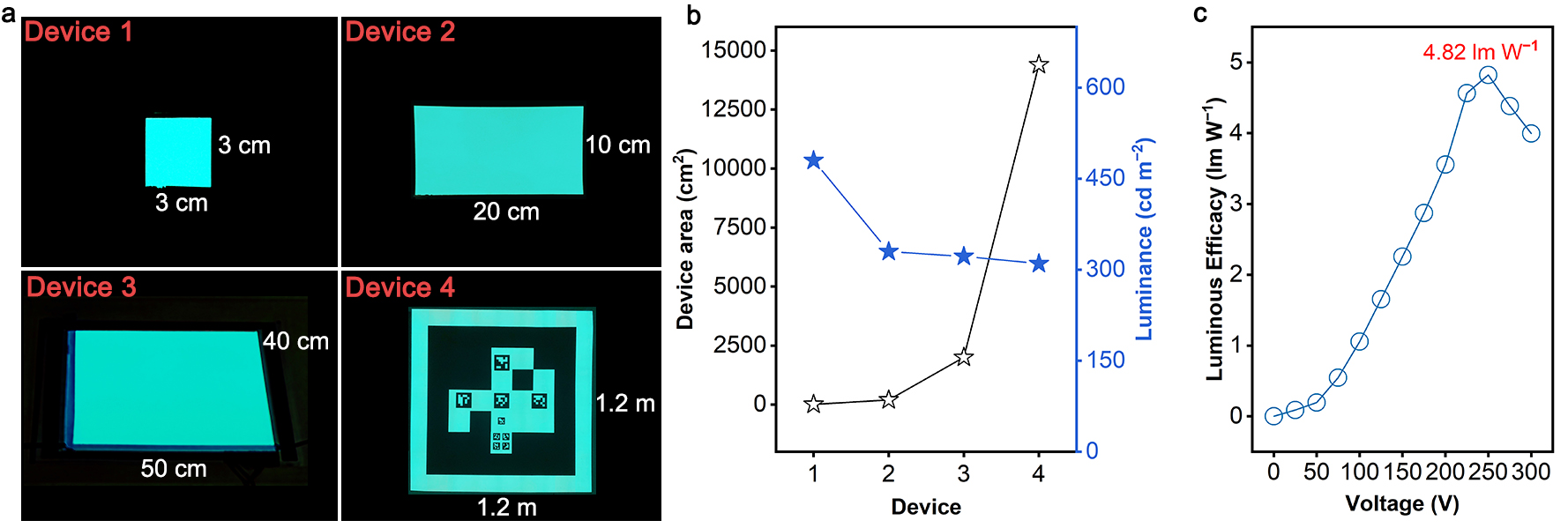


**Figure S12.** (a) Photographs of ACEL devices with various luminescent areas ranging from 9 cm^2^ to 14400 cm^2^. (b) Statistical chart of luminous area and brightness for different devices. Notably, the small-sized devices reach a maximum brightness of nearly 500 cd m^−2^, while the ultra-large device with 1.2 m × 1.2 m area maintains a remarkable brightness of 303.3 cd m^−2^. (c) Luminous efficacy as a function of AC voltage for device 1. A maximum luminous efficacy was recorded as 4.82 lm W^−1^.


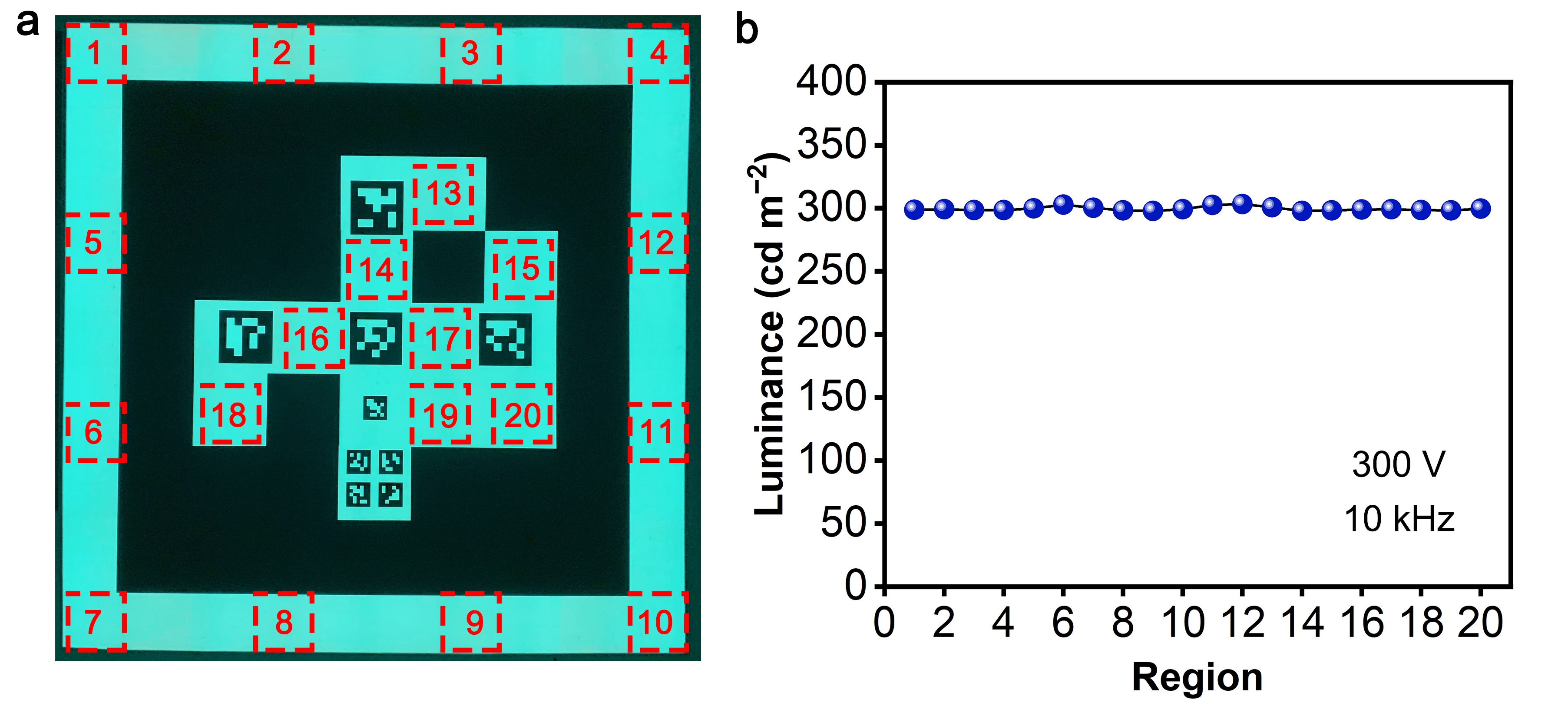


**Figure S13.** (a) Photograph of the large-scale ACEL device, highlighting 20 selected representative test points. (b) Statistical chart of luminance measurements at the 20 detection points. All brightness readings are approximately 300 cd cm^−2^, as measured with a handheld brightness meter placed on the surface of the device.

**S10. Simulation of electric field distribution.**


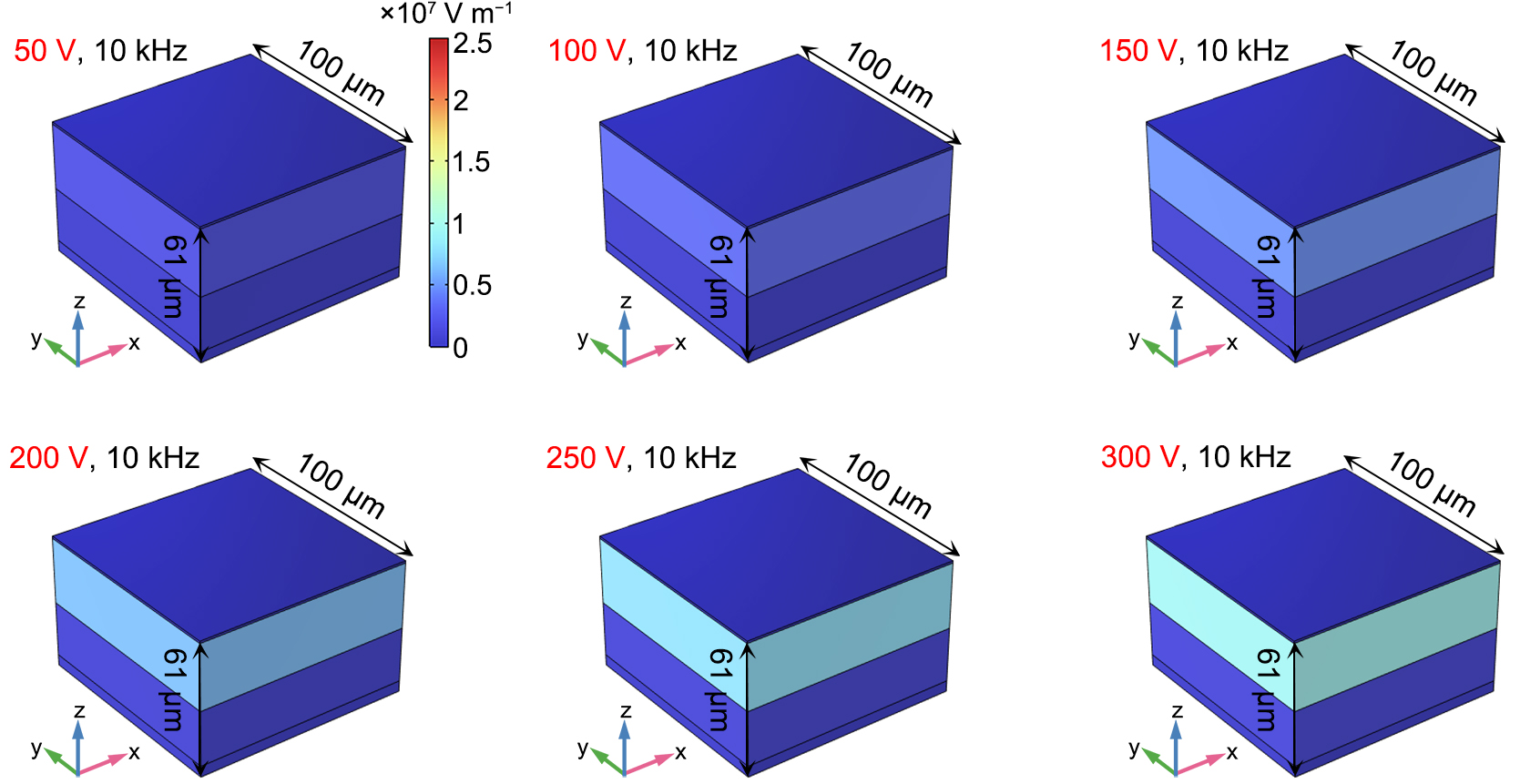


**Figure S14.** Simulations of drive voltage dependent electric field distribution. The simulation was conducted under a fixed driving frequency of 10 kHz, with the AC voltage varied from 50 V to 300 V. Correspondingly, the electric field intensity within the luminescent layer increased from 0.3×10^7^ V m^−1^ to 1.2×10^7^ V m^−1^ as the driving voltage rose. The enhanced electric field significantly increases the number of luminescent centers transitioning from the ground state to the excited state, thereby improving the material's electron-hole recombination probability and enhancing the device's electroluminescent intensity. This simulation result provides a theoretical basis for the experimental observation of voltage-dependent EL brightness, confirming that adjusting the driving voltage is an effective strategy to regulate the electric field distribution and thus optimize the luminescent performance of ACEL devices.


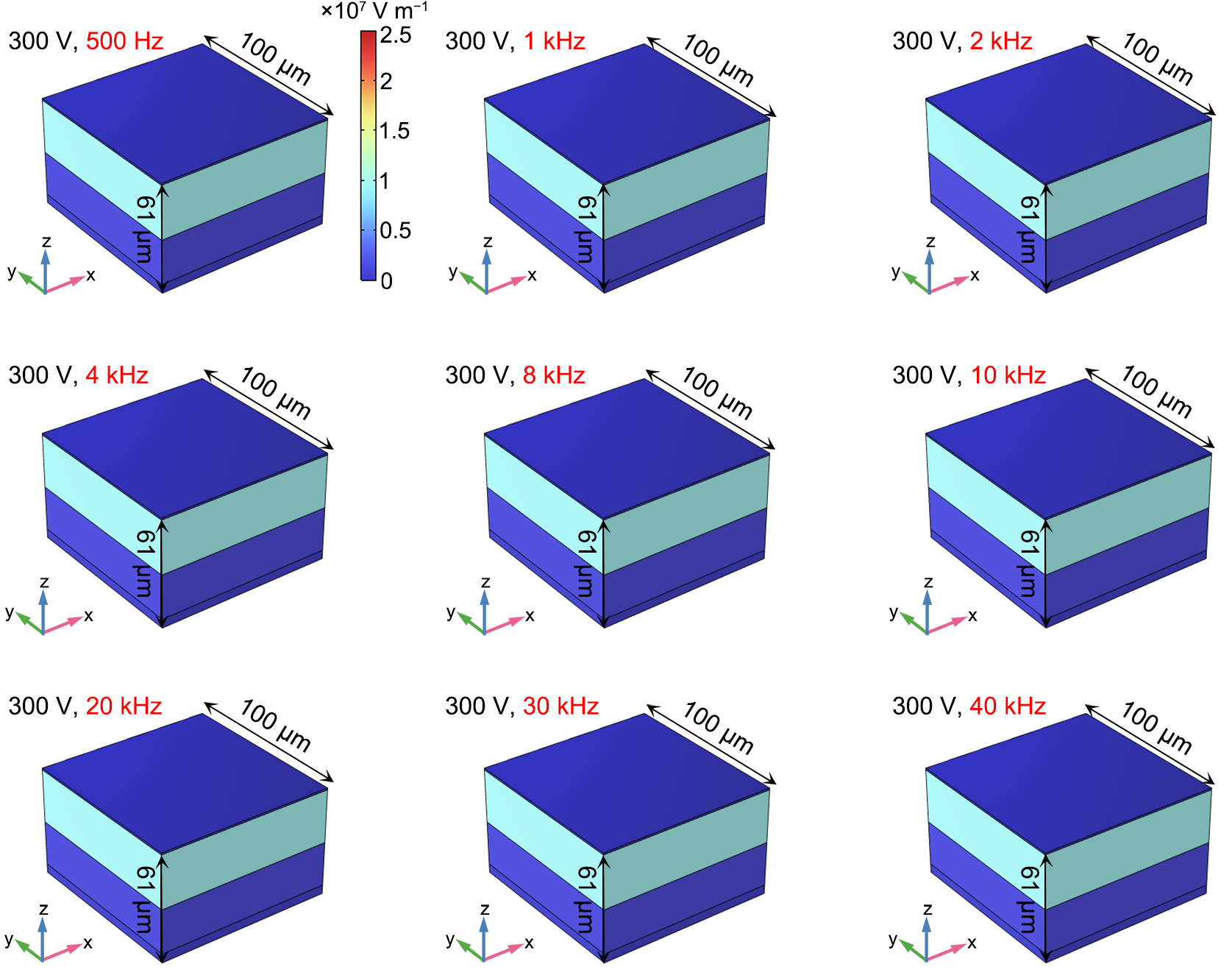


**Figure S15.** Simulations of drive frequency dependent electric field distribution. The simulation was performed under a fixed AC voltage of 300 V, with the driving frequency ranging from 500 Hz to 40 kHz. Simulation results indicate that the electric field intensity within the luminescent layer remained unchanged as the driving frequency increased over the tested range. In contrast, experimental observations indicate that even with a constant voltage, altering the driving frequency still leads to a significant increase in brightness. This is primarily due to the higher frequency alternating electric field, which more effectively excites charge transitions and recombination in the ZnS:Cu phosphor.

**S11. Performance comparison of reported ACEL devices**

**Table S4. Comparison of ACEL device performance**

| Year | Emitting layer (EL) | Dielectric layer (DL) | Process | Structure | Performance | Maximum Luminance | Luminescent Area | Ref. |
| --- | --- | --- | --- | --- | --- | --- | --- | --- |
| 2024 | ZnS:Cu | BaTiO_3_ | Spin coating | PDMS/ conductive polymer (SACP)/EL&DL/SACP/EL&DL/SACP/PDMS | Stretchable EL; Maintain ≈81% of their initial luminance after 1000 times of repeated disassembly and reassembly. | L_max_ > 300 cd m^−2^ (300 V, 32 kHz) | 25 mm^2^ | 5 |
| 2024 | ZnS:Cu  @Perovskite | PDMS/SEBS | Spray coating and spin coating | ITO/EL&DL/Ag;  SEBS/AgNWs/EL&DL/AgNWs/SEBS | Stretchable EL; Expands the color gamut of powder-based ACEL devices by 250% using perovskite color-conversion. | L_max_ > 500 cd m^−2^ (200 V, 20 kHz) | 2.56 cm^2^ | 6 |
| 2020 | ZnS:Mn/ZnS:Cu | SBS/PVDF-HFP | Spin coating and blade coating | PET/ITO/EL&DL/AgNWs | High mechanical robustness; Color tunable (orange/white/blue). | L_max_ = 45 cd m^−2^ (250 V, 2 kHz) | 3 cm^2^ | 7 |
| 2024 | ZnS:Cu | BQ10 | Screen-printing | PET/Ag/DL/EL/PEDOT:PSS | Sustainability with plant-based resin matrices. | L_max_ = 30 cd m^−2^ (220 V, 2 kHz) | 3.14 cm^2^ | 8 |
| 2016 | ZnS:Cu/Mo/Cl | acrylic elastomer (VHB 4910) | Multi-layer stacking process | Al/ionic conductors/VHB/EL/VHB/ionic conductors/Al | Stretchable EL; Transparency | L_max_ = 10 cd m^−2^ (5 kV, 1 kHz) | 4 cm^2^ | 9 |
| 2022 | ZnS:Cu,Cl | PDMS | Spin coating | PDMS/CuNWs/EL&DL/CuNWs | Stretchable EL; The first demonstration of CuNWs applied in a stretchable ACEL. | L_max_ = 97.6 cd m^−2^ (170 V, 60 kHz) | 4 cm^2^ | 10 |
| 2024 | ZnS:Cu | BaTiO_3_/PVDF-HFP | Spray coating | TPU/EGaIn/DL/EL/AgNWs/TPU | Repeatable machine washing; Direct manufacturing on textiles. | L_max_ = 338.6 cd m^−2^ (188 V, 5 kHz) | 9 cm^2^ | 11 |
| 2025 | ZnS:Cu | BaTiO_3_/TPU | Electrospinning and spray coating | RCNM/EGaIn/EL&DL/AgNWs/PDMS | Combination of passive radiative cooling and active electroluminescence. | L_max_ > 80 cd m^−2^ (180 V, 16 kHz) | 12 cm^2^ | 12 |
| 2024 | ZnS:Cu | BaTiO_3_/PU | Spin coating | AgNWs/PU/EL&DL/Silver paste | Devices are stable after 50 bend-release cycles. | L_max_ = 50.6 cd m^−2^ (300 V, 100 Hz) | 16 cm^2^ | 13 |
| 2022 | ZnS:Cu | BaTiO_3_/Ecoflex | Scraping coating | ITO/EL&DL/Silver;  Ecoflex/Ag Microflake/EL&DL/Hydrogel | Biaxial tensile area strains up to 750%; Multiple encryption with dynamic pattern switching. | L_max_ > 100 cd m^−2^ (250 V, 64 kHz) | 18 cm^2^ | 14 |
| 2023 | ZnS:Cu | BaTiO_3_/TPU | Hot pressed and blade coating | Ag&TPU/EL&DL/Compo-SiL/TPU | Displays colored  and pixelated patterns and dynamic images. | L_max_ = 70 cd m^−2^ (120 V, 50 kHz) | 30 cm^2^ | 15 |
| 2024 | ZnS:Cu@Al_2_O_3_ | WPU | Hot pressed and blade coating | ITO/PET/WPU&EL/CNT&WPU/PET | Reflection-emission hybrid mode is proposed; Low power consumption. | L_max_ = 107.5 cd m^−2^ (200 V, 40 kHz) | 168 cm^2^ | 16 |
| 2021 | ZnS:Cu/Mn | TPU | Melt-spinning and dip-coated | silver-plated nylon yarns//EL&DL/ionicliquid-doped &TPU | Integrating displays into textiles; Brightness between  EL units deviates by less than 8%. | L_max_ = 122 cd m^−2^ (370 V, 2 kHz) | 1.5 m^2^ | 17 |
| 2025 | ZnS:Cu | BaTiO_3_ | Aerosol spray | PET/Cu paste/DL/EL/PETDOT:PSS | Substrate compatibility and flexibility; Exceptional operational stability | L_max_ = 303.3 cd m^−2^ (300 V, 10 kHz) | 1.44 m^2^ | **This work** |
| 2025 | ZnS:Cu | BaTiO_3_ | Aerosol spray | PET/Cu paste/DL/EL/PETDOT:PSS | Substrate compatibility and flexibility; Exceptional operational stability | L_max_ > 500 cd m^−2^ (300 V, 10 kHz) | 9 cm^2^ | **This work** |
| 2021 | MAPbI_3_ | —— | Blade-coating | ITO/poly-TPD/Perovskite/TPBi/LiF/Al | Large-Area PeLED; Achieves 16.1% EQE via low-temperature blade-coating and sol-gel engineering. | L_max_ > 30 W Sr^−1^ m^−2^ (5 V, DC) | 28 cm^2^ | 18 |
| 2025 | CsPbBr_3_ | —— | Blade-coating | ITO/Poly-TPD/PVK/2PACz/Perovskite/TPBi/LiF/Al | FAAc boosts perovskite nucleation; Interlayer anchors nuclei, slows growth, improves crystal quality. | L_max_ = 36000 cd m^−2^ (6 V, DC) | 35 cm^2^ | 19 |
| 2022 | CsPb(Br_x_Cl_1−x_)_3_ (0 < x < 1) | —— | Blade-coating | ITO/PVK/Perovskite/TPBi/LiF/ Al | Blade-coating supersaturated CsPb(Br₀.₈₄Cl₀.₁₆)₃ solution yields uniform films with better optoelectronic properties. | L_max_ = 1500 cd m^−2^ (6V, DC) | 28 cm^2^ | 20 |
| 2025 | CsPbBr_3_ | —— | Spin coating | ITO/PEDOT:PSS/HTMs/Perovskite/TPBi/LiF/Al | Small-molecule HTM X15 with SMe boosts Pe-QLED efficiency to 21.10% (1 cm²), outperforming PTAA. | L_max_ = 13066 cd m^−2^ (4.5 V, DC) | 1 cm^2^ | 21 |
| 2025 | FPEA_2_SnI_4_ | —— | Spin coating | ITO/m-PEDOT: PSS/Perovskite/TPBi/LiF/Al | γ acid enables stable, large-area Sn perovskite PeLEDs with high luminance and quantum efficiency. | L_max_ = 371 cd m^−2^ (4 V, DC) | 2.25 m^2^ | 22 |
| 2020 | FAPbBr_3_ | —— | Spin coating | TPD/PEDOT:PSS/Poly-TPDTBB/Perovskite/TPBI/LiF/Al | A molecular interface strategy enables green PeLEDs with 20.1% EQE and 20×20 mm devices over 16% EQE. | L_max_ = 5980 cd m^−2^ (4.5 V, DC) | 4 cm^2^ | 23 |

**Table S4** and **Figure S16** present a statistical analysis comparing the luminance performance of the ACEL devices developed in this work with those related reports based on inorganic luminescent particles and perovskite nanocrystals^5-23^, focusing on four key performance and fabrication parameters: (1) fabrication method, (2) maximum luminance, (3) driving conditions (voltage/frequency), and (4) luminescent area. Detailed numerical values for these parameters are summarized in Table S4 for direct quantitative reference. Notably, the meter-scale flexible ACEL devices fabricated via a low-cost, simple aerosol spraying method using the modified ZnS:Cu-based luminescent in exhibit notable advantages over reported counterparts: they maintain high luminance > 300 cd m^−2^ even at meter-scale luminescent areas, outperforming most previously reported ACEL devices that either show reduced luminance at large areas or are limited to centimeter-scale sizes. This fabrication strategy thus offers a practical, scalable approach to advancing high-brightness, large-area flexible display and solid-state lighting technologies, addressing the critical challenge of balancing luminescent performance, dimensional scalability, and fabrication simplicity in traditional ACEL device development.


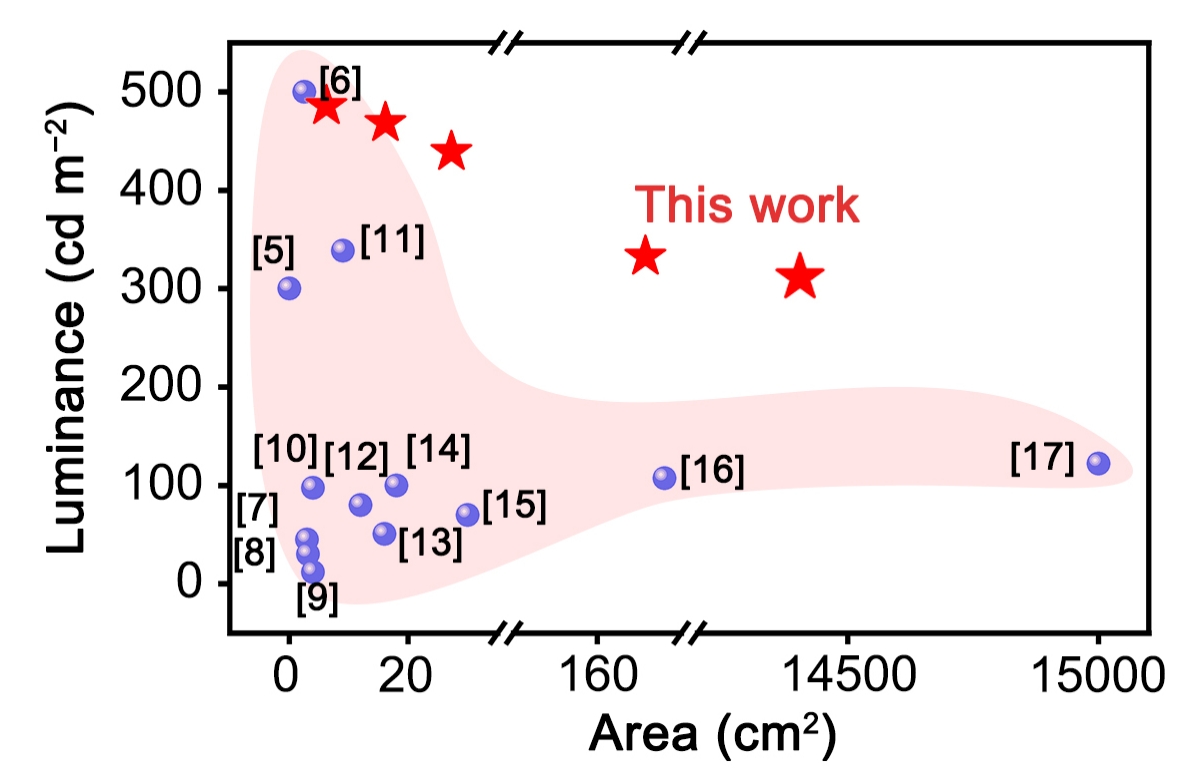


**Figure S16.** Statistical comparison of area-dependent luminance performance of ACEL devices.

**S12. Supplementary videos**

Supplementary Video 1. The third-person perspective of drone operation

Supplementary Video 2. The drone-camera perspective in drone operation

**References**

1. C. Lin, R. Yang, Z. Wu, Y. Wang, C. Zhan, H. Miyata, X. Zeng, Y. Zheng, S. You, Y. Lv, Y. Zhuang, R.-J. Xie, J. Ueda, Electrically chargeable inorganic persistent luminescence in an alternating current driven electroluminescent device. *Commun. Mater.* **2025**, *6*, 25.
2. Z. Liu, H. Yang, H. Yuan, Y. Cheng, B. Xu, M. Xue, T. Jing, Full-color, highly bright and stretchable electroluminescent device with janus colors based on photoluminescent electrode for wireless dynamical display. *Composites Part B* **2024**, *286*, 111787.
3. P. Dang, G. Li, S. Liang, H. Lian, J. Lin, Multichannel photoluminescence tuning in Eu-doped apatite phosphors via coexisting cation substitution, energy transfer and valence mixing. *J. Mater. Chem. C* **2019**, *7*, 5975.
4. D. Park, W. Kim, C. Park, J. Choi, A. Ghorai, G. Lee, S. Choi, W. Moon, U. Jeong, Interactive deformable colored sound display achieved with electrostrictive fluoropolymer and halide perovskite. *Small* **2024**, *20*, 2402281.
5. Z. Luo, W. Chen, M. Lai, S. Shi, P. Chen, X. Yang, Z. Chen, B. Wang, Y. Zhang, X. Zhou, Fully printable and reconfigurable Hufu‐type electroluminescent devices for visualized encryption. *Adv. Mater.* **2024**, *36*, 2313909.
6. F. Chun, B. Zhang, Y. Gao, X. Wei, Q. Zhang, W. Zheng, J. Zhou, Y. Guo, X. Zhang, Z. Xing, X. Yu, F. Wang, Multicolour stretchable perovskite electroluminescent devices for user-interactive displays. *Nat. Photonics* **2024**, *18*, 856.
7. Y. Zuo, X. Shi, X. Zhou, X. Xu, J. Wang, P. Chen, X. Sun, H. Peng, Flexible color‐tunable electroluminescent devices by designing dielectric‐distinguishing double‐stacked emissive layers. *Adv. Funct. Mater.* **2020**, *30*, 2005200.
8. R. Polícia, N. Peřinka, C. Mendes-Felipe, P. Martins, D. M. Correia, S. Lanceros-Méndez, Toward sustainable electroluminescent devices for lighting and sensing. *Adv. Sustain. Syst.* **2024**, *8*, 2400140.
9. C. H. Yang, B. Chen, J. Zhou, Y. M. Chen, Z. Suo, Electroluminescence of giant stretchability. *Adv. Mater.* **2015**, *28*, 4480.
10. P. Tran, N.-H. Tran, J.-H. Lee, Highly stretchable electroluminescent device based on copper nanowires electrode. *Sci. Rep.* **2022**, *12*, 8967.
11. Y. Zhang, X. Wang, Y. Zhang, M. Liu, Z. Zhao, Z. Shen, Y. Hu, Wearable alternating current electroluminescent e‐textiles with high brightness enabled by fully sprayed layer‐by‐layer assembly. *Adv. Funct. Mater.* **2023**, *34*, 2308969.
12. Y. Gong, Y. Ma, J. Xing, X. Wang, M. Liu, Y. Zhang, S. Xu, D. Li, Y. Xiong, Z. Shen, Y. Hu, High-performance nanomembranes integrating radiative cooling and alternating current luminescence for smart wearable. *Chem. Eng. J.* **2025**, *505*, 159214.
13. X. Zhang, C. Liu, J. Shan, Z. Li, X. Guo, X. Zhao, J. Ding, H. Yang, Flexible electroluminescent device based on ZnS:Cu-polyurethane composite and silver nanowires. *J. Mater. Sci. - Mater. Electron.* **2024**, *35*, 123.
14. B. Yang, Y. Zhao, M. U. Ali, J. Ji, H. Yan, C. Zhao, Y. Cai, C. Zhang, H. Meng, Asymmetrically enhanced coplanar‐electrode electroluminescence for information encryption and ultrahighly stretchable displays. *Adv. Mater.* **2022**, *34*, 2201342.
15. Y. Lin, X. Chen, Q. Lu, J. Wang, C. Ding, F. Liu, D. Kong, W. Yuan, W. Su, Z. Cui, Thermally laminated lighting textile for wearable displays with high durability. *ACS Appl. Mater. Interfaces* **2023**, *15*, 5931.
16. J. Shi, Q. Fan, S. Zhu, Y. Gu, G. Liu, T. Zhou, W. Chen, F. Xiong, L. Peng, L. Li, Z. Qin, S. Deng, B. R. Yang, Dual‐mode flexible electrophoretic e‐paper with integration of alternating current electroluminescent technology for ubiquitous ambient light applications. *Adv. Funct. Mater.* **2024**, *34*, 2410139.
17. X. Shi, Y. Zuo, P. Zhai, J. Shen, Y. Yang, Z. Gao, M. Liao, J. Wu, J. Wang, X. Xu, Q. Tong, B. Zhang, B. Wang, X. Sun, L. Zhang, Q. Pei, D. Jin, P. Chen, H. Peng, Large-area display textiles integrated with functional systems. *Nature* **2021**, *591*, 240.
18. S. Chu, W. Chen, Z. Fang, X. Xiao, Y. Liu, J. Chen, J. Huang, Z. Xiao, Large-area and efficient perovskite light-emitting diodes via low-temperature blade-coating. *Nat. Commun.* **2021**, *12*, 147.
19. G. Chen, S. Wang, Z. Yu, C. Dong, P. Jia, D. Pu, K. Dong, H. Cui, H. Fang, C. Wang, R. Gao, F. Yao, W. Ke, G. Li, G. Fang, Regulation of nucleation and crystallization for blade-coating large-area CsPbBr3 perovskite light-emitting diodes. *Science Bulletin* **2025**, *70*, 212.
20. S. Chu, Y. Zhang, P. Xiao, W. Chen, R. Tang, Y. Shao, T. Chen, X. Zhang, F. Liu, Z. Xiao, Large-Area and Efficient Sky-Blue Perovskite Light-Emitting Diodes via Blade-Coating. *Adv. Mater.* **2022**, *34*, 2108939.
21. X. Li, H. Ahangar, S. Yang, J. Huang, E. Sheibani, A. V. Kuklin, X. Luo, F. A. Ghahfarokhi, C. Wei, H. Ågren, G. Baryshnikov, B. Xu, Defect Passivating Hole Transporting Material for Large-Area and Stable Perovskite Quantum-Dot Light-Emitting Diodes. *ACS Nano* **2025**, *19*, 6784.
22. J. Wang, D. Han, B. Zhao, Z. Zang, H. Ji, L. Liu, N. Wang, Push-Pull Effect Enables Large-Area Lead-Free Perovskite Light-Emitting Diodes via Electron Directional Transfer. *Laser & Photonics Reviews* **2025**, *19*, e00727.
23. H. Wang, X. Gong, D. Zhao, Y.-B. Zhao, S. Wang, J. Zhang, L. Kong, B. Wei, R. Quintero-Bermudez, O. Voznyy, Y. Shang, Z. Ning, Y. Yan, E. H. Sargent, X. Yang, A Multi-functional Molecular Modifier Enabling Efficient Large-Area Perovskite Light-Emitting Diodes, *Joule* **2020**, *4*, 1977.
